# Transcriptome analysis reveals a positive effect of brassinosteroids on the photosynthetic capacity of wucai under low temperature

**DOI:** 10.1186/s12864-019-6191-2

**Published:** 2019-11-06

**Authors:** Mengru Zhao, Lingyun Yuan, Jie Wang, Shilei Xie, Yushan Zheng, Libing Nie, Shidong Zhu, Jinfeng Hou, Guohu Chen, Chenggang Wang

**Affiliations:** 1 College of Horticulture, Vegetable Genetics and Breeding Laboratory, Anhui Agricultural University, 130 West Changjiang Road, Hefei, 230036 Anhui China; 2 Provincial Engineering Laboratory for Horticultural Crop Breeding of Anhui, 130 West of Changjiang Road, Hefei, 230036 Anhui China; 3 Wanjiang Vegetable Industrial Technology Institute, Maanshan, 238200 Anhui China

**Keywords:** RNA-Seq, Low-temperature stress, Photosynthesis, Porphyrin and chlorophyll metabolism

## Abstract

**Background:**

Brassinosteroids (BRs) have a positive effect on many processes during plant growth and development, and in response to various abiotic stressors. Low-temperature (LT) stress constricts the geographic distribution, growth, and development of wucai (*Brassica campestris* L. ssp. *chinensis* var. *rosularis* Tsen). However, there is little information on the global gene expression of BRs under LT stress in wucai. In this study, the molecular roles of 24-epibrassinolide (EBR) after exogenously application, were explored by RNA sequencing under LT conditions.

**Results:**

According to the Gene Ontology (GO) term and Kyoto Encyclopedia of Genes and Genomes (KEGG) pathway analyses, photosynthesis was significantly enriched after spraying EBR under LT. The transcripts encoding the photosystem II (PSII) oxygen-evolving enhancer protein, photosystem I (PSI) subunit, light-harvesting chlorophyll protein complexes I and II, and ferredoxin were up-regulated after the application of EBR. Transcripts encoding several key enzymes involved in chlorophyll biosynthesis were also up-regulated, accompanied by significant differences in the contents of 5-aminolevulinic acid (ALA), porphobilinogen (PBG), protoporphyrin IX (Proto IX), Mg-protoporphyrin IX (Mg-proto IX), protochlorophyllide (Pchl), and photosynthetic pigments. Notably, transcriptional and physiological analyses revealed that under LT stress, plant responses to EBR involved a major reorientation of photosynthesis, as well as porphyrin and chlorophyll metabolism.

**Conclusion:**

This study explored the role of EBR as an LT stress tolerance mechanism in wucai. At the transcription level, LT tolerance manifests as an enhancement of photosynthesis, and the amelioration of porphyrin and chlorophyll metabolism.

## Background

Wucai (*Brassica campestris* L. ssp. *chinensis* var. *rosularis* Tsen) is a variant of non-heading Chinese cabbage (*Brassica campestris* L.), a crucial species in the Brassicaceae family [1]. As an important autumn and winter vegetable crop, wucai is cultured throughout most parts of China, especially in the Yangtze-Huaihe River Basin. With a beautiful shape and high levels of 70 vitamins and minerals, wucai is becoming an increasingly popular crop in other countries.

Brassinosteroids (BRs) were isolated from *Brassica* pollen ~ 40 years ago, and they are a type of plant steroid hormone. 24-epibrassinolide (EBR), one of the BRs, is biologically-active brassinolides hormones used in physiological and molecular studies [2]. Moreover, previous studies found that BRs regulate a variety of physiological and developmental processes, including plant stress tolerance, leaf elongation, and flower development, which up-regulate large genes involved in cell division and differentiation, and exhibit control over all developmental processes [3]. BRs are known to regulate photosynthesis under normal as well as in abnormal conditions [4, 5]. The studies of Choudhary et al. [6] and Yuan et al. [7] showed that foliar application of EBR can increase plant chlorophyll (Chl) content and up-regulate the expression levels of various oxidative stress marker genes, thereby improving photosynthetic capacity to enhance the tolerance of *Arabidopsis* and *Cucumis sativus* L. responding to different stresses.

Low-temperature (LT) stress is harmful to numerous plant physiological processes and is the main abiotic stressor that limits plant growth and development. For example, LT stress can stunt plant growth, disrupt photosynthesis, and reduce chlorophyll content, resulting in significant yield and economic losses. Recently, our team reported that the differentially expressed proteins in wucai (*Brassica campestris* L.) produced by temperature stress were mainly enriched in porphyrin and chlorophyll metabolism, carbon metabolism, carbon fixation in photosynthetic organisms, and so on [8]. Previous research demonstrated that the exogenous application of BRs significantly improved resistance to LT stress by regulating the morphological, physiological, and biochemical characteristics of pepper (*Capsicum annuum* L.) [9, 10], tomato (*Solanum lycopersicum* Mill.) [11], and cucumber (*Cucumis sativus* L.) [12]. Transcriptome analysis indicated that the DEGs of Winter Rapeseed (*Brassica rapa* L.) under cold stress were significantly enriched in plant hormone signal transduction, starch and sucrose metabolism, and photosynthesis [13]. Jaglo illustrated that the freezing tolerance of *B. napus* can be enhanced through CBF-mediated “regulon engineering” [14]. The results of these studies indicate that exogenous applications of BRs reduce the adverse effects of LT stress by increasing chlorophyll content, maintaining photosynthesis, and activating gene expression and signal transduction pathways [15, 16].

**Fig. 1:**
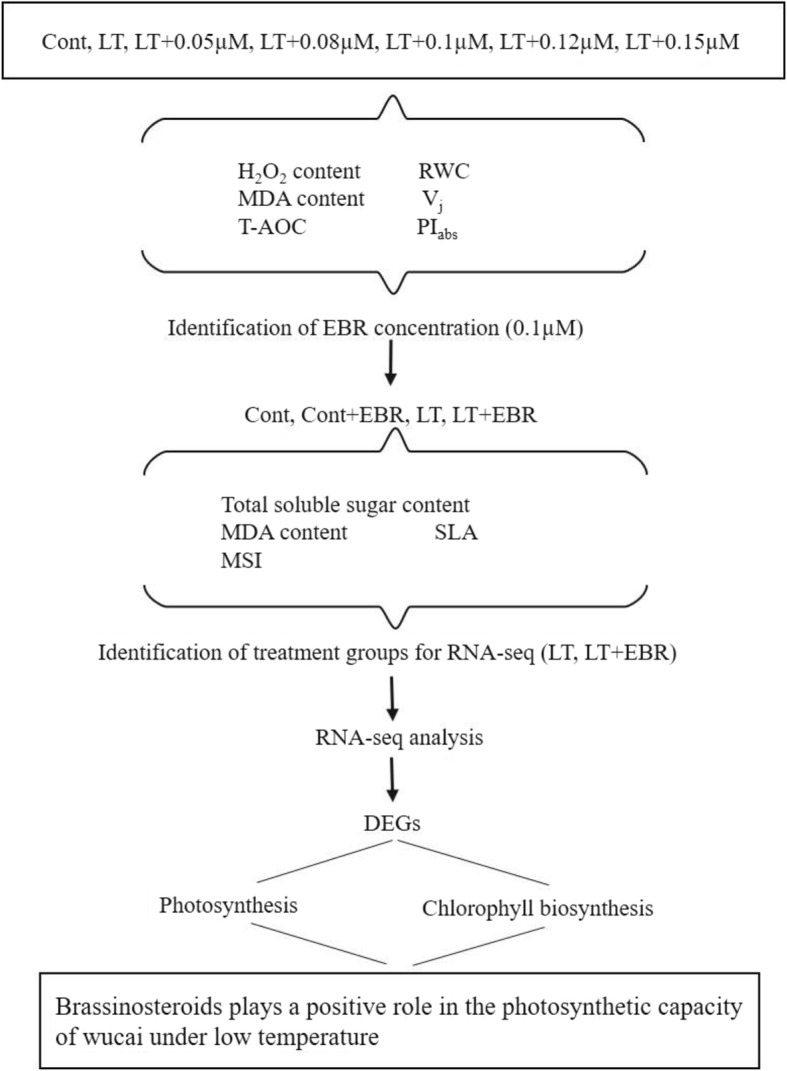
Experimental design ideas

RNA-Seq technology is a high-throughput transcriptome analysis platform that can expeditiously, accurately, and economically explore instantaneous gene expression of multiple, diverse, and even non-model species [17]. In this study, RNA-Seq analysis was conducted in order to explore how gene expression correlates with LT stress and the signaling pathways regulated by 24-epibrassinolide (EBR). A physiological analysis of chlorophyll biosynthesis and photosystem activity was also conducted in order to identify a key pathway for transcriptional regulation (Fig. 1). The goals of this study were to: (1) elucidate the transcriptomic response to EBR and LT stress; (2) reveal the genes and pathways that correlate with EBR-induced cold tolerance; and, (3) determine the interactive influences of EBR and LT stress on leaf photosystem activity and chlorophyll biosynthesis in wucai. The findings of this work will elucidate the underlying mechanisms of this plant growth regulator regulation and the response mechanisms of no-heading cabbage species to LT stress.

## Results

### Physiological parameter effects of EBR

**Fig. 2:**
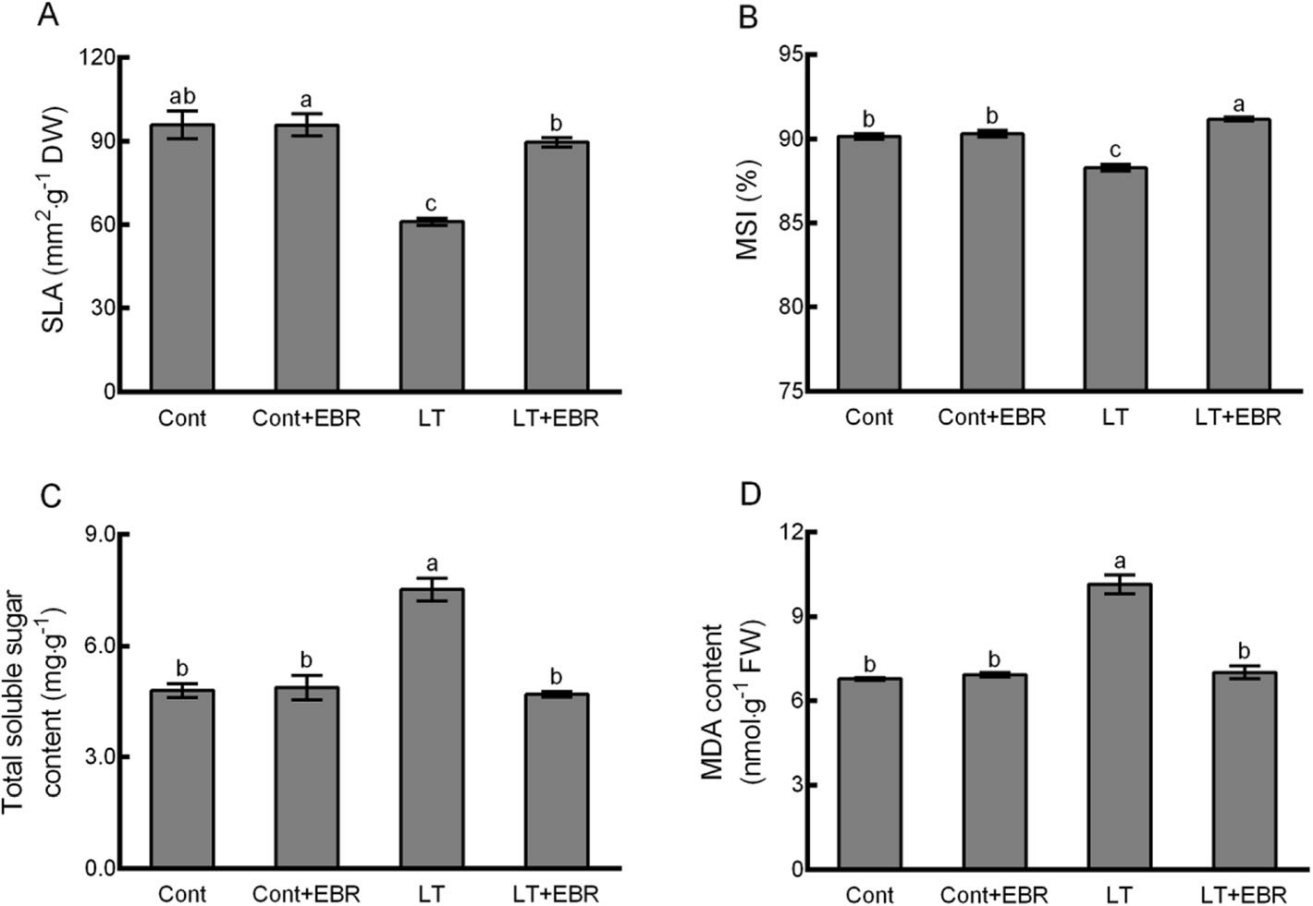
Effects of exogenous EBR application on SLA (**a**), MSI (**b**), total soluble sugar content (**c**), and MDA (**d**) in wucai leaves under LT stress. The data are represented as the means ± standard error (SE). Bars with different letters above the columns indicate significant differences (*p* < 0.05, Duncan’s range test) on a given day of treatment

The physiological parameters reflecting plant cold injury (e.g., specific leaf area (SLA), membrane stability index (MSI), total soluble sugar content, and malondialdehyde (MDA)) were not significantly different between spraying with EBR (Cont+EBR) and the control (Cont). When subjected to LT stress, spraying EBR (LT + EBR) led to significant differences in SLA (Fig. 2a), MSI (Fig. 2b), total sugar content (Fig. 2c), and MDA (Fig. 2d) content compared to LT plants. Temperature had a significant effect and all 4 of the parameters were significantly different between LT and Cont. There were no significant differences in SLA, total soluble sugar content, and MDA between LT + EBR and Cont (Fig. 2).

The largest change was in the total soluble sugar content among the 4 indicators. Compared to Cont, the total soluble sugar content increased by 56.87% in LT plants (Fig. 2c). Furthermore, the total soluble sugar content of LT plants significantly decreased after spraying EBR under LT stress (37.59%, Fig. 2c). The MDA content followed a similar trend. Compared to Cont, MDA content significantly increased by 49.81% in LT plants, and significantly decreased by 30.95% in LT + EBR (Fig. 2d). For the LT treatments specifically, SLA was depressed by 36.33%. Compared to LT, SLA significantly increased by 46.80% in LT + EBR plants (Fig. 2a). Compared to Cont, the MSI content of LT was significantly reduced by 2.07%*.* In contrast, MSI significantly increased by 1.14% after EBR treatment (Fig. 2b). Compared to LT, SLA significantly increased by 3.27% in LT + EBR (Fig. 2a).

### Mapping and quantitative assessment of Illumina sequences

Two libraries were constructed from the LT and LT + EBR treatment groups for analysis by RNA-Seq. A total of 53.51 million (LT) and 50.83 million (LT + EBR) reads were generated. After removing the adapter, poly-N, low-quality, and empty reads, > 6 billion clean bases were obtained with a Q30 percentage > 94%, and a GC percentage between 47.4–47.7% (Additional file 5: Table S1). The quality control (fastqc v0.11.5, Illumina) and reads mapping (fastqc v0.11.5, Illumina) were shown in Additional file 1: Figure S1 and Additional file 2: Figure S2.

Each library of clean reads was aligned to the *Brassica rapa* (*B. rapa*) reference genome [18]. The proportion of clean reads in the 2 wucai transcriptome libraries that mapped to the *B. rapa* reference genome ranged from 86.67–87.56% (Additional file 6: Table S2). A total of 30,843 genes were confirmed from the mapped libraries. All of the RNA- sequence data in this article have been deposited in the NCBI- SRA database and are accessible in SRP200451 (https://www.ncbi.nlm.nih.gov/sra/SRP200451).

### Transcriptome profiles

**Fig. 3:**
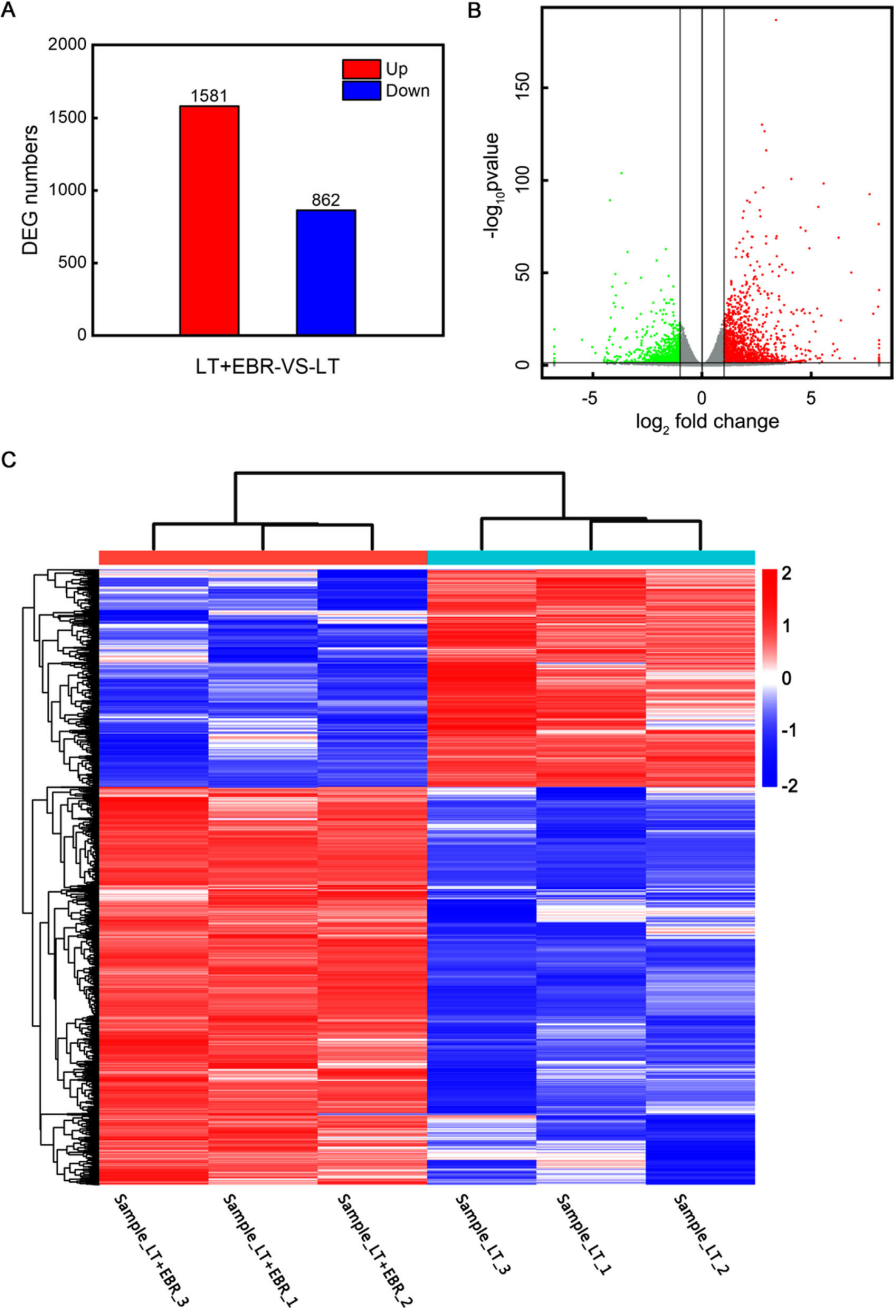
Transcriptome analysis of the DEGs in the LT and LT + EBR treatments of wucai leaves. **a** Comparisons of DEGs in the LT and LT + EBR treatments. **b** Volcano plot showing the DEGs between two different libraries. The threshold *q* < 0.05 was used to determine the significance of DEGs. Red and green dots represent up- and down-regulated genes, respectively, and gray dots indicate transcripts that did not change significantly in the LT + EBR library compared to LT. **c** Hierarchical clustering of all of the DEGs was based on the log_10_RPKM values. The color spectrum from blue to red represents the gene expression intensity from low to high, respectively

The 30,843 genes from the mapped libraries were normalized using the reads per kilo bases per million reads (RPKMs) method [19]. A total of 2443 differentially expressed genes (DEGs) were identified between LT and LT + EBR (Fig. 3a, b). Hierarchical clustering of all of the DEGs was conducted to observe the gene expression patterns and was evaluated by the log_10_RPKMs of the 2 groups (Fig. 3c). Compared to LT, LT + EBR contained 1581 up-regulated and 862 down-regulated genes. These results suggest that EBR had a marked effect on the transcription of a subset of genes in response to LT stress.

### GO and KEGG analyses of the EBR-induced DEGs

**Fig. 4:**
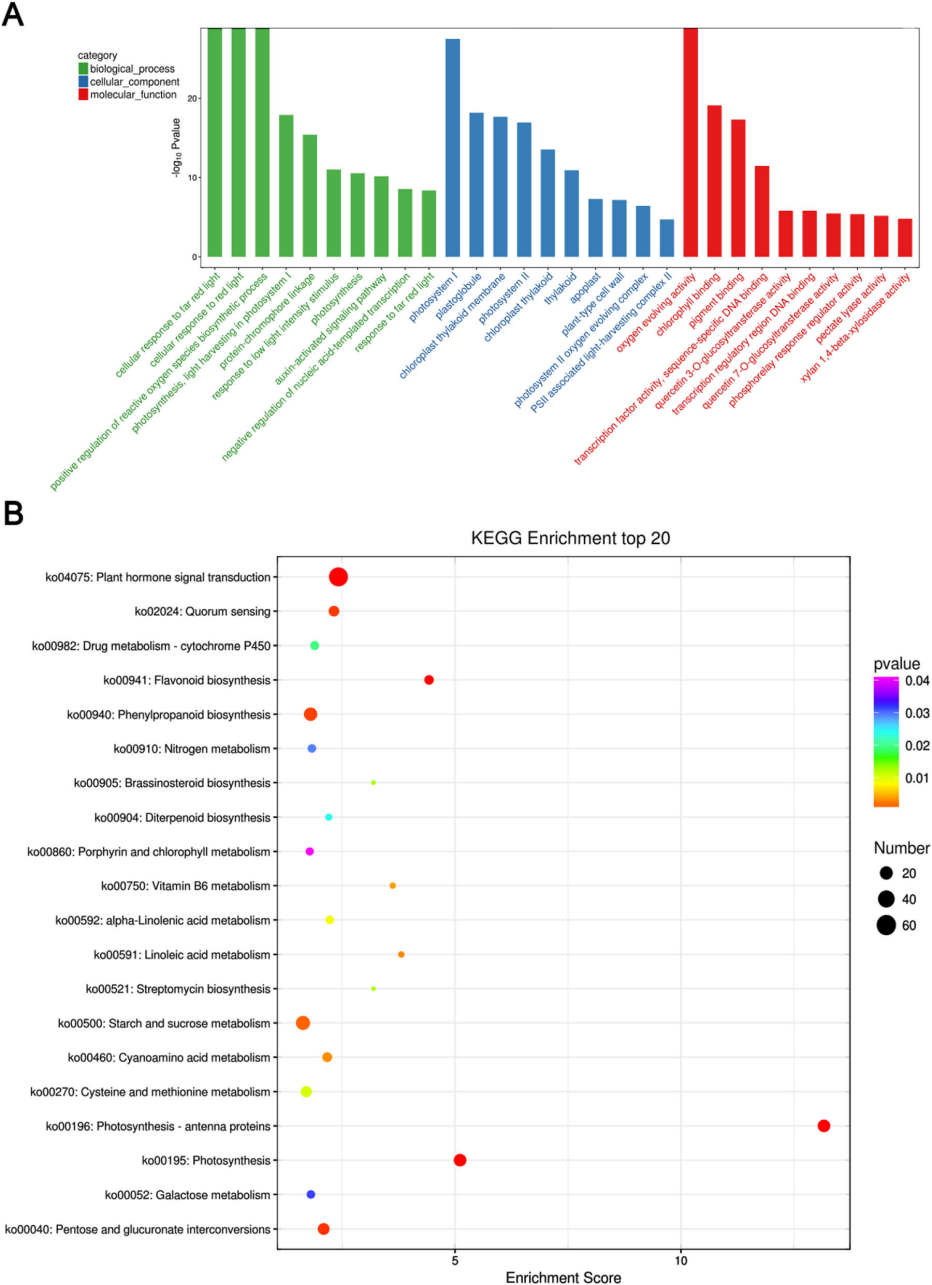
GO and KEGG pathway enrichment analyses of the DEGs in the LT and LT + EBR treatments of wucai leaves. **a** GO enrichment analysis with the 30 most enriched GO terms in the 3 categories shown. **b** KEGG enrichment analysis with the 20 most enriched KEGG terms shown. High and low *p*-values are represented by blue and red, respectively

In order to explore the function of the EBR-induced DEGs under LT stress, the DEGs were used in the Gene Ontology (GO) enrichment analysis. Results uncovered the biological process, molecular function, and cellular component categories for the 2443 DEGs of the 2 groups (Fig. 4a). The transcriptome analysis sifted GO terms with the number of DEGs > 2 in the 3 classifications, according to the corresponding -log_10_Pvalue of each term; 10 terms were sorted from large to small. In the biological process category, cellular response to far red light (GO:0071490) was the most abundant, followed by cellular response to red light (GO:0071491), indicating the positive regulation of reactive oxygen species biosynthetic processes (GO:1903428), such as photosynthesis and light harvesting in photosystem I (GO:0009768). Similarly, in the cellular component category, photosystem I (GO:0009522) was the most abundant, followed by plastoglobule (GO:0010287), chloroplast thylakoid membrane (GO:0009535), and photosystem II (GO:0009523). In the molecular function category, oxygen-evolving activity (GO:0010242) was the most abundant, followed by chlorophyll binding (GO:0016168), pigment binding (GO:0031409), transcription factor activity, and sequence-specific DNA binding (GO:0003700). Besides, the expression patterns of the cold acclimation, cold-induced and BR-responsive genes were listed in Additional file 7: Table S3 and Additional file 8: Table S4.

Among the DEGs, there were 7 pathways with Kyoto Encyclopedia of Genes and Genomes (KEGG) annotations that were significantly affected: (1) photosynthesis–antenna proteins, (2) photosynthesis, (3) plant hormone signal transduction, (4) flavonoid biosynthesis, (5) pentose and glucuronate interconversions, (6) quorum sensing, and (7) phenylpropanoid biosynthesis (*p* < 0.001; Fig. 4b). Higher enrichment scores and more genes were observed in photosynthesis–antenna proteins (24 genes) and photosynthesis (24 genes).

### Quantitative real-time PCR (qRT-PCR) analysis

**Fig. 5:**
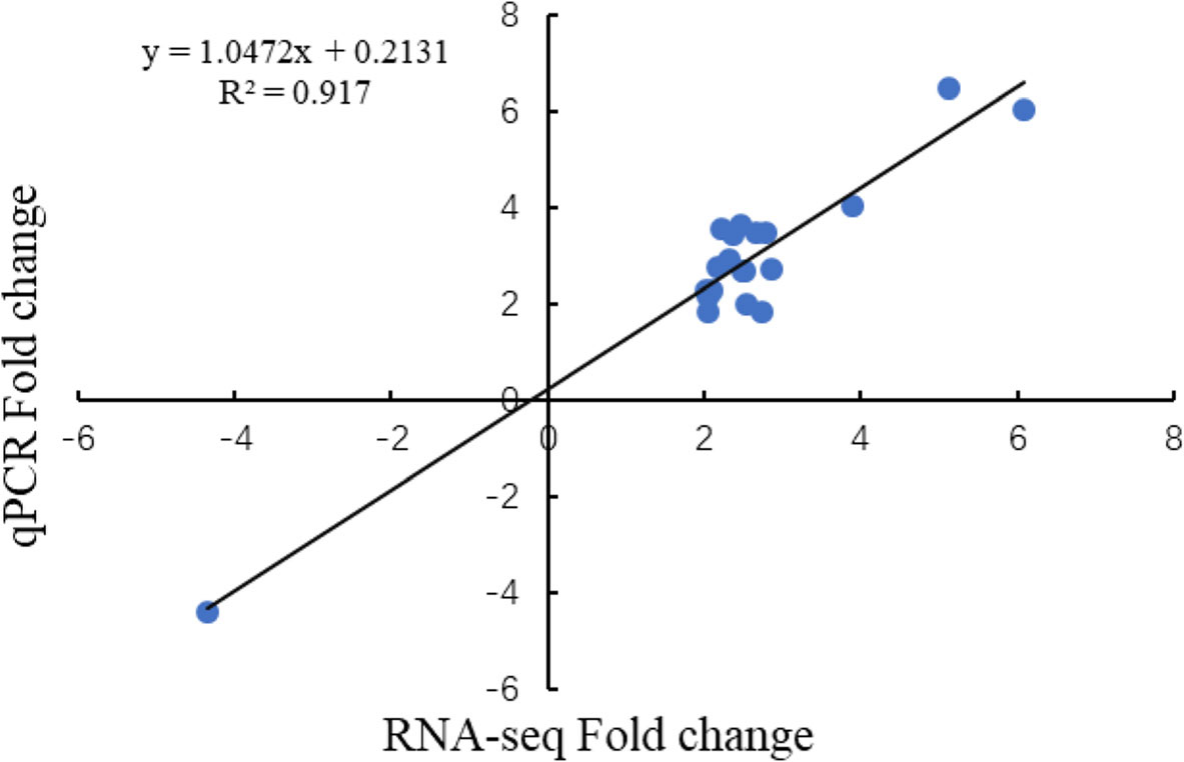
Correlation of RNA-Seq (x-axis) qRT-PCR data (y-axis). The assay was carried out for 20 randomly selected DEGs. GO and KEGG enrichment analyses

In order to validate the DEG data from RNA-Seq, 20 DEGs were randomly selected for quantitative real-time (qRT)-PCR assays in EBR-mediated LT stress (Additional file 3: Figure S3). Results indicated a strong positive correlation with the RNA-Seq data (R^2^ = 0.917), thereby validating the RNA-Seq data (Fig. 5; Additional file 9: Table S5).

### Effects of EBR on Porphyrin and chlorophyll metabolism analysis

**Fig. 6:**
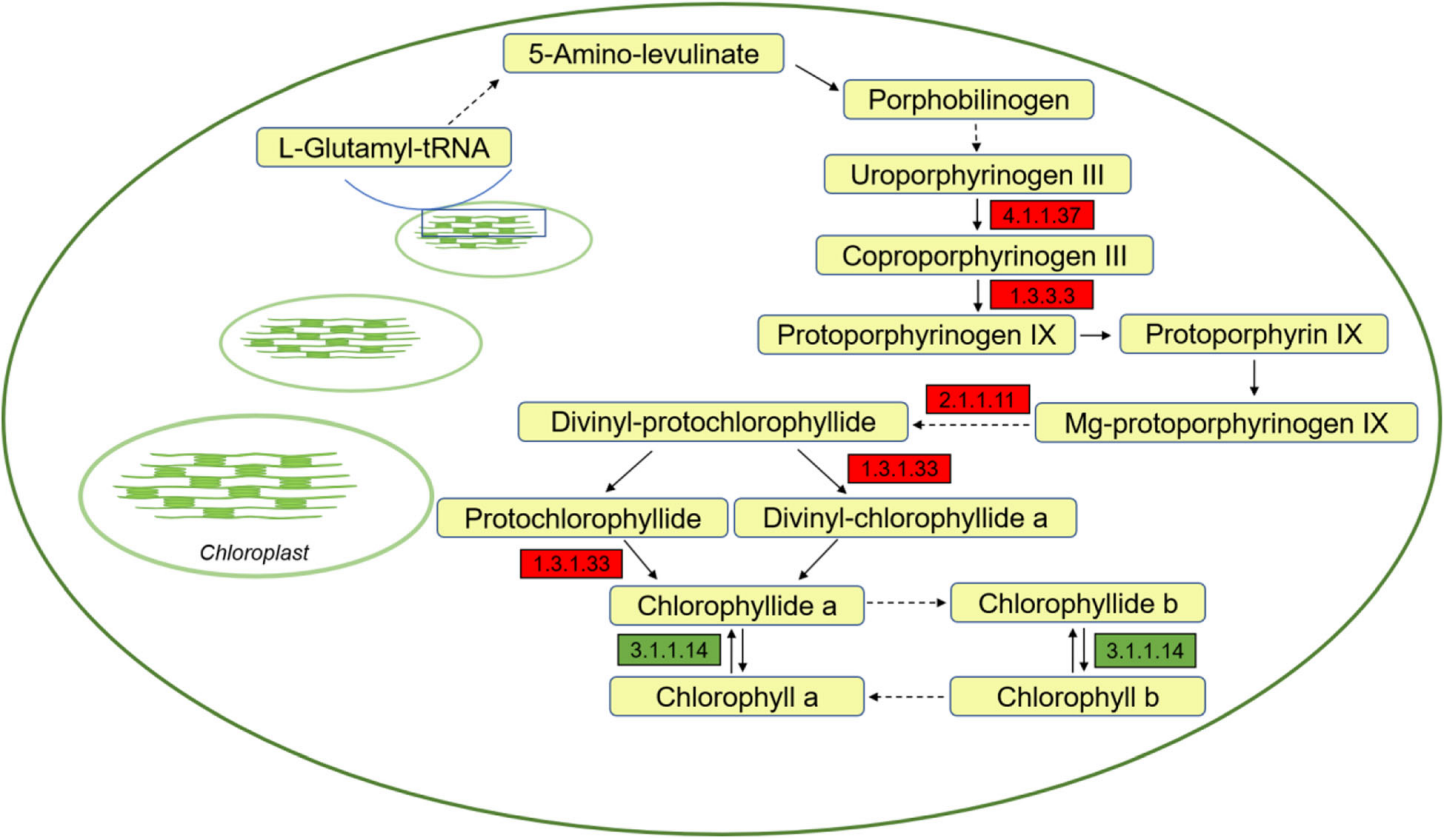
Porphyrin and chlorophyll metabolism-related gene expression in wucai leaves influenced by LT stress alone or in combination with exogenous EBR application. Red and green represent the up- and down-regulation of genes, respectively. Up-regulated genes encode uroporphyrinogen decarboxylase (EC:4.1.1.37), coproporphyrinogen III oxidase (EC:1.3.3.3), magnesium-protoporphyrin O-methyltransferase (EC:2.1.1.11), and protochlorophyllide reductase (EC:1.3.1.33), while the down-regulated gene encodes chlorophyllase (EC:3.1.1.14)

From the KEGG pathway analysis, results revealed that an exogenous EBR pretreatment can up-regulate the expression of several genes in porphyrin and chlorophyll metabolic pathways under LT conditions compared to Cont (Fig. 6). These up-regulated genes encode enzymes that cover almost the whole process of 5-aminolevulinic acid (ALA) and chlorophyll biosynthesis, including uroporphyrinogen decarboxylase (EC:4.1.1.37), coproporphyrinogen III oxidase (EC:1.3.3.3), magnesium-protoporphyrin O-methyltransferase (EC:2.1.1.11), and protochlorophyllide reductase (EC:1.3.1.33). The down-regulated gene encodes chlorophyllase (EC:3.1.1.14).

**Fig. 7:**
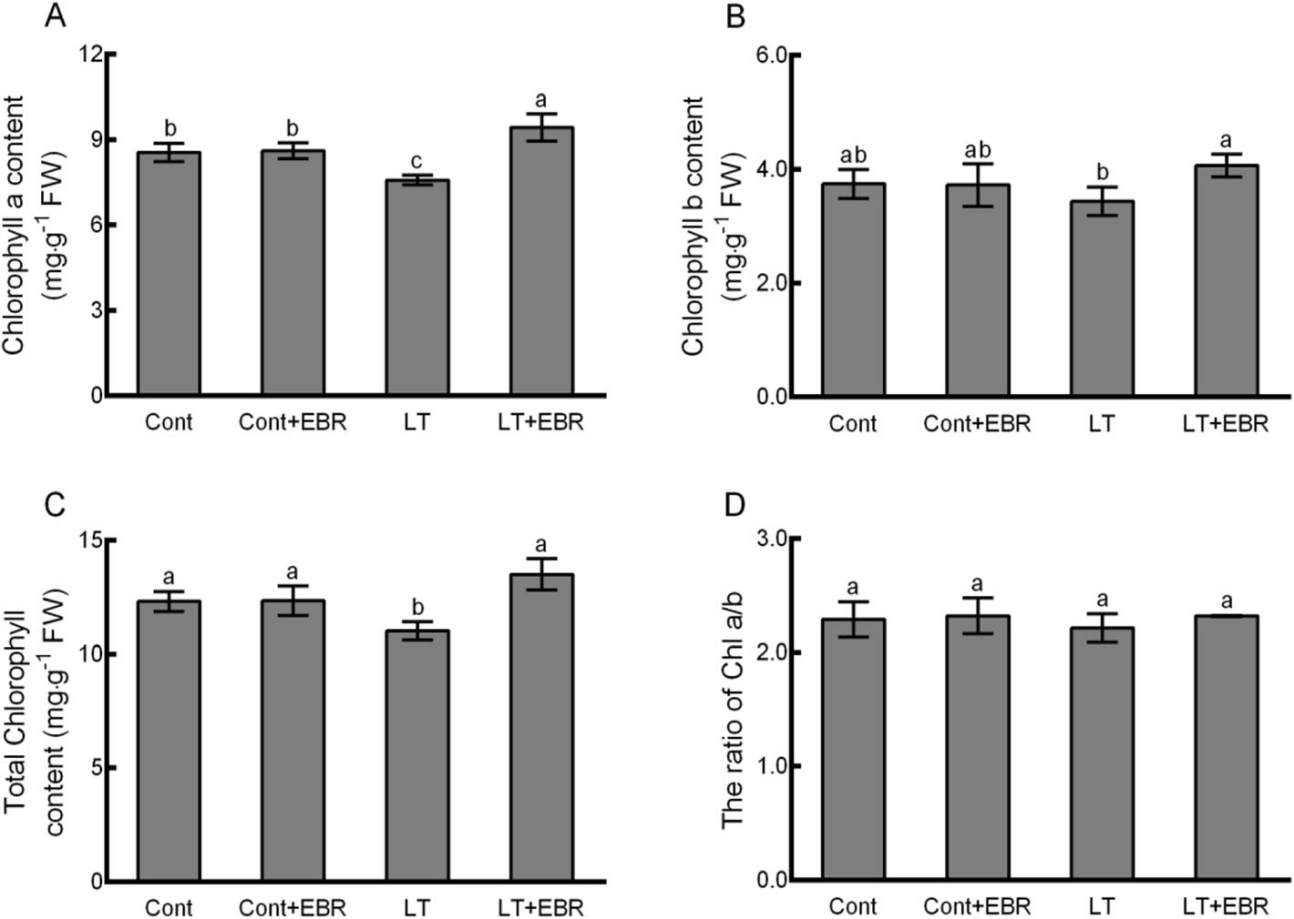
Effects of exogenous EBR application on photosynthetic pigment contents in wucai leaves under LT stress. (A–C) Quantification of the Chl *a* content (**a**), Chl *b* content (**b**), Total Chl content (**c**), and the Chl *a*/*b* ratio calculated from A and B (**d**). The data are presented as the mean ± SE. Bars with different letters above the columns indicate significant differences (*p* < 0.05, Duncan’s range test) on a given day of treatment

**Fig. 8:**
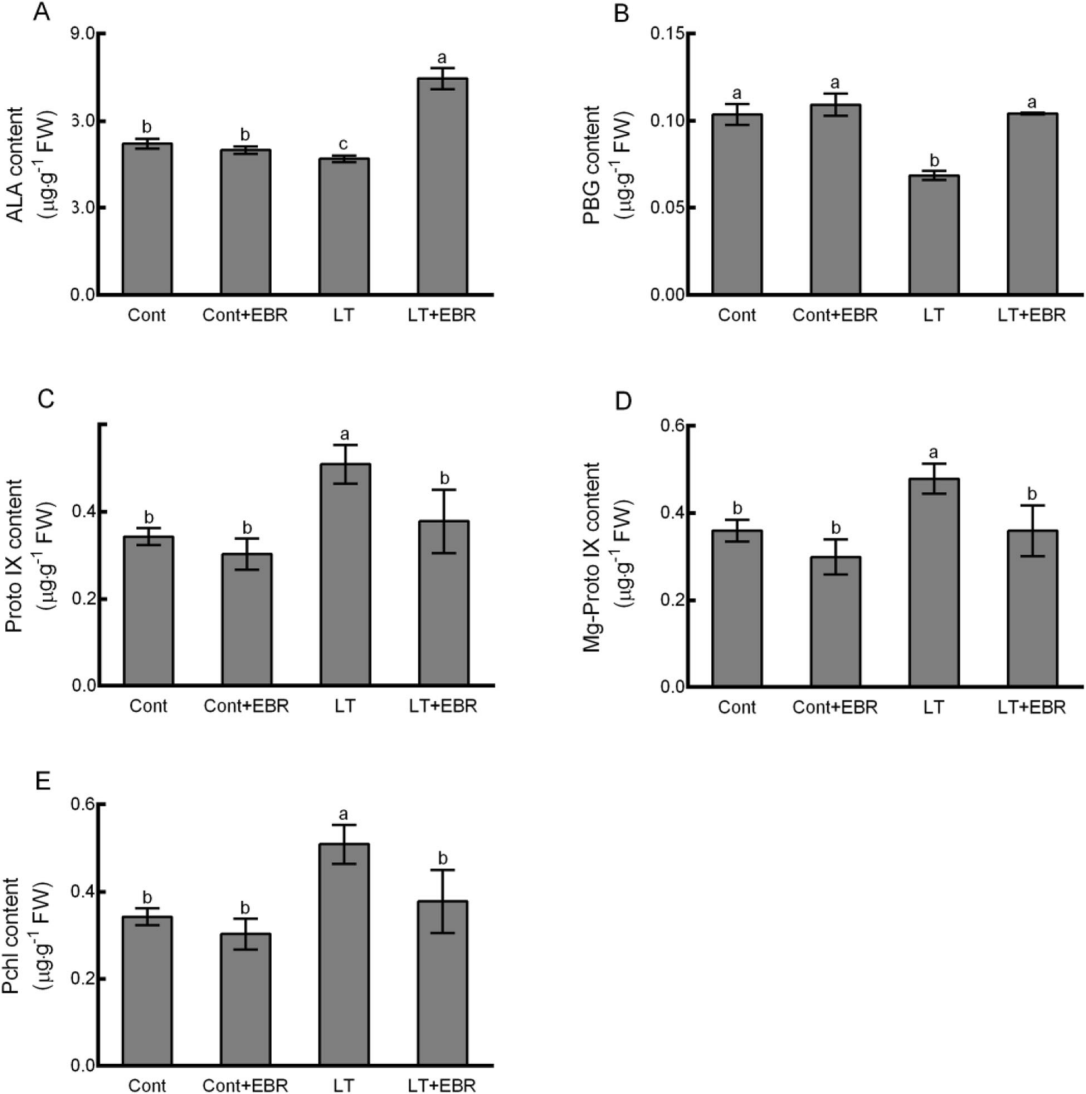
Effects of exogenous EBR application on porphyrin and chlorophyll metabolism in wucai leaves under LT stress. Quantification of ALA content (**a**), PBG content (**b**), Proto IX content (**c**), Mg-Proto IX content (**d**) and Pchl content (**e**). The data are presented as the mean ± SE. Bars with different letters above the columns indicate significant differences (*p* < 0.05, Duncan’s range test) on a given day of treatment

In order to determine whether exogenous EBR treatment influenced the photosynthetic pigment contents and metabolite levels involved in porphyrin and chlorophyll metabolism, the contents of chlorophyll *a* (Chl *a*), chlorophyll *b* (Chl *b*), total chlorophyll (Total Chl) content, the Chl *a*/*b* ratio, ALA, porphobilinogen (PBG), protoporphyrin IX (Proto IX), Mg-protoporphyrin IX (Mg-Proto IX), and protochlorophyllide (Pchl) were assessed. No significant differences were detected between Cont and Cont+EBR (Figs. 7 and 8). Significant increases in Chl *a*, Chl *b*, Total Chl content, ALA, and PBG were detected in LT + EBR plants after 5 d compared to LT, with increases of 24.44, 18.41, 22.56, 59.01, and 52.06%, respectively. In contrast, the contents of Proto IX, Mg-Proto IX, and Pchl significantly declined by 25.45, 24.97, and 25.78%, respectively, when LT + EBR was compared to LT. Additionally, LT resulted in significant declines in Chl *a*, Total Chl content, ALA, and PBG by 11.41, 10.47, 10.04, and 33.89%, respectively, when LT was compared to Cont. Conversely, significant increases by 34.25, 33.22, and 48.58% were noted in the Proto IX, Mg-Proto IX, and Pchl contents, respectively, when LT was compared to Cont. There were no significant differences detected between LT + EBR and Cont/Cont+EBR, except inthe Chl *a* and ALA contents.

### Effect of EBR on photosynthesis

**Fig. 9:**
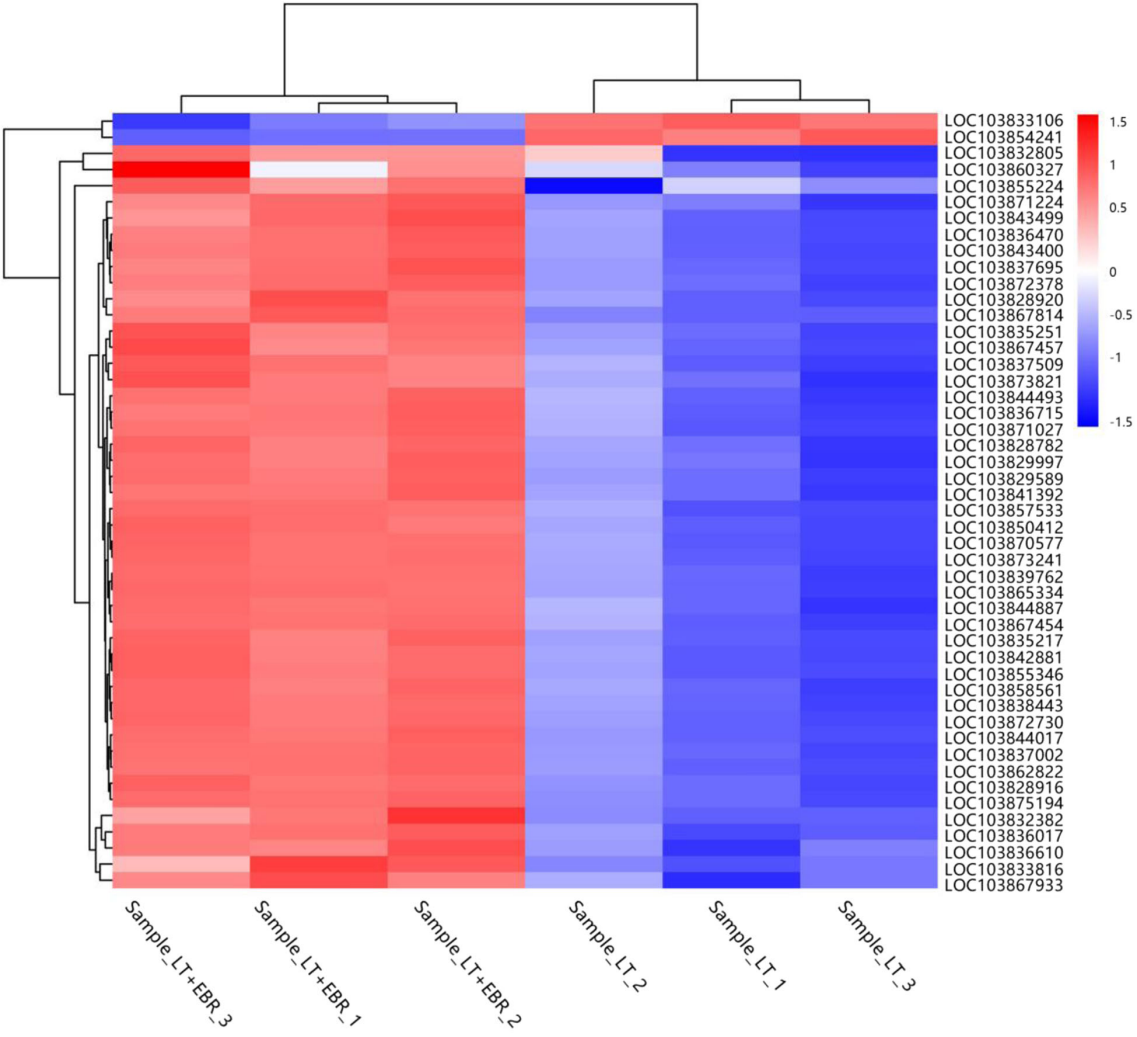
Heat map of the photosynthesis and photosynthesis-antenna proteins-related DEGs in the LT and LT + EBR treatment of wucai leaves. The log_2_FoldChange (FPKM) values were used to generate the heat maps using MeV software. Red and blue represent high or low expression levels, respectively, than those shown in white

**Fig. 10:**
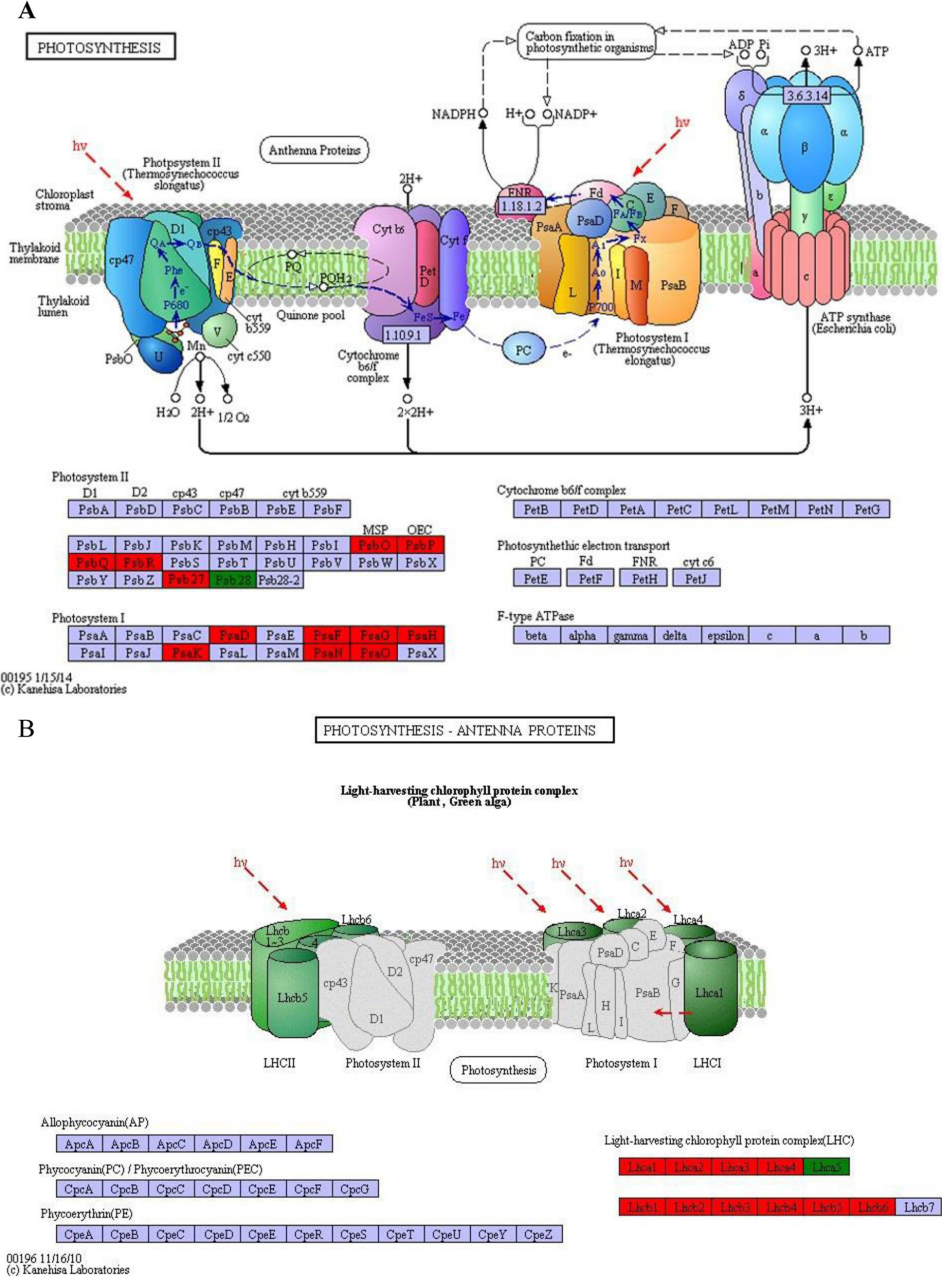
Photosynthesis-related (A) and photosynthesis-antenna proteins-related (B) gene expression in wucai leaves influenced by LT alone or in combination with exogenous EBR application based on the KEGG pathway analysis. Red, green, and blue represent the up-, down-, and non-regulation of genes

**Fig. 11:**
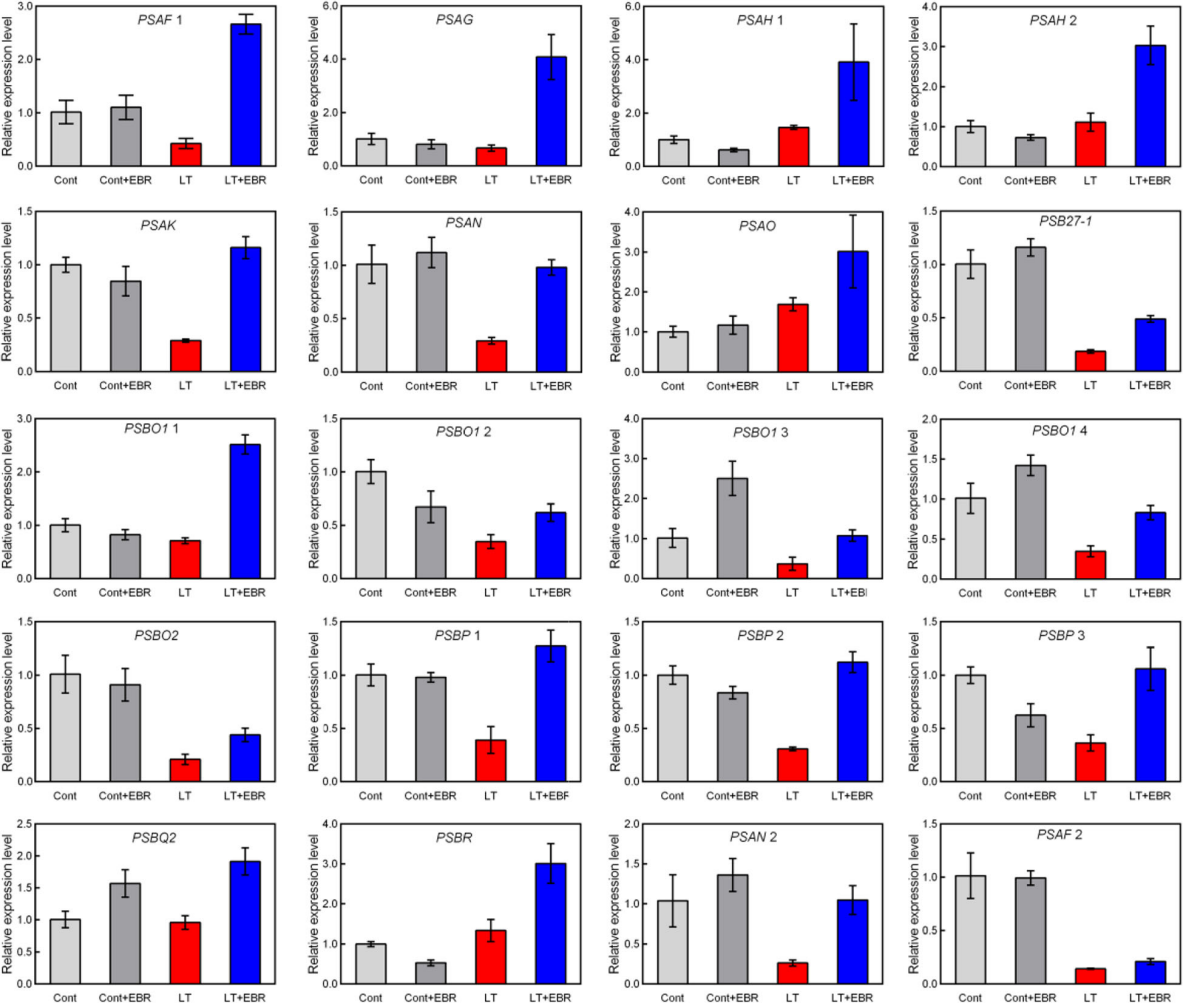
Effects of exogenous EBR application on photosynthesis-related genes in wucai leaves under LT stress

In LT + EBR plants, the genes involved in photosynthesis were primarily up-regulated (Fig. 9), with a focus on PSII, PSI, and the light-harvesting chlorophyll protein complex (LHC) (Fig. 10a, b). In PSII, the DEGs included oxygen-evolving enhancer protein 1 (Psb O), oxygen-evolving enhancer protein 2 (Psb P), oxygen-evolving enhancer protein 3 (Psb Q), 10 kDa protein (Psb R), and Psb27 protein (Psb 27). In PSI, the DEGs included subunit II (Psa D), subunit III (Psa F), subunit V (Psa G), subunit VI (Psa H), subunit X (Psa K), and subunits Psa N and Psa O. The chlorophyll a/b-binding protein was also up-regulated, which is well-known as an important component of the light-harvesting chlorophyll protein complexes I and II [20]. In total, 23 genes encoding the chlorophyll a/b-binding protein were enhanced by EBR application (Fig. 11, Additional file 10: Table S6, Additional file 11: Table S7).

**Fig. 12:**
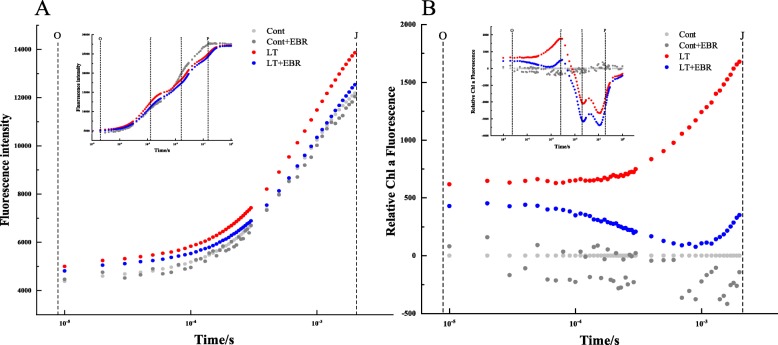
Fast Chl *a* fluorescence transient (OJIP) plotted on logarithmic time scale (0.00001–1 s) measured under Cont, Cont+EBR, LT, and LT + EBR (**a**), and the calculated relative Chl *a* fluorescence transient (**b**)

**Fig. 13:**
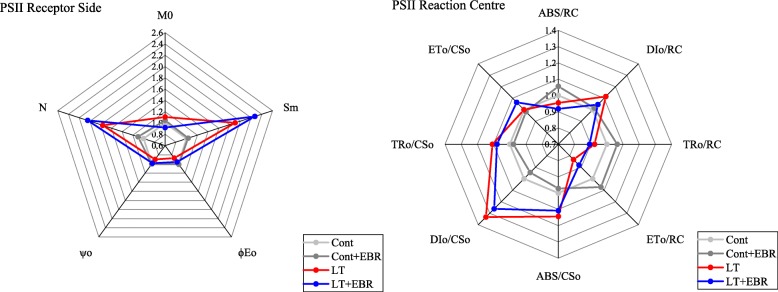
Effects of exogenous EBR application on PSII in wucai leaves under LT stress

**Fig. 14:**
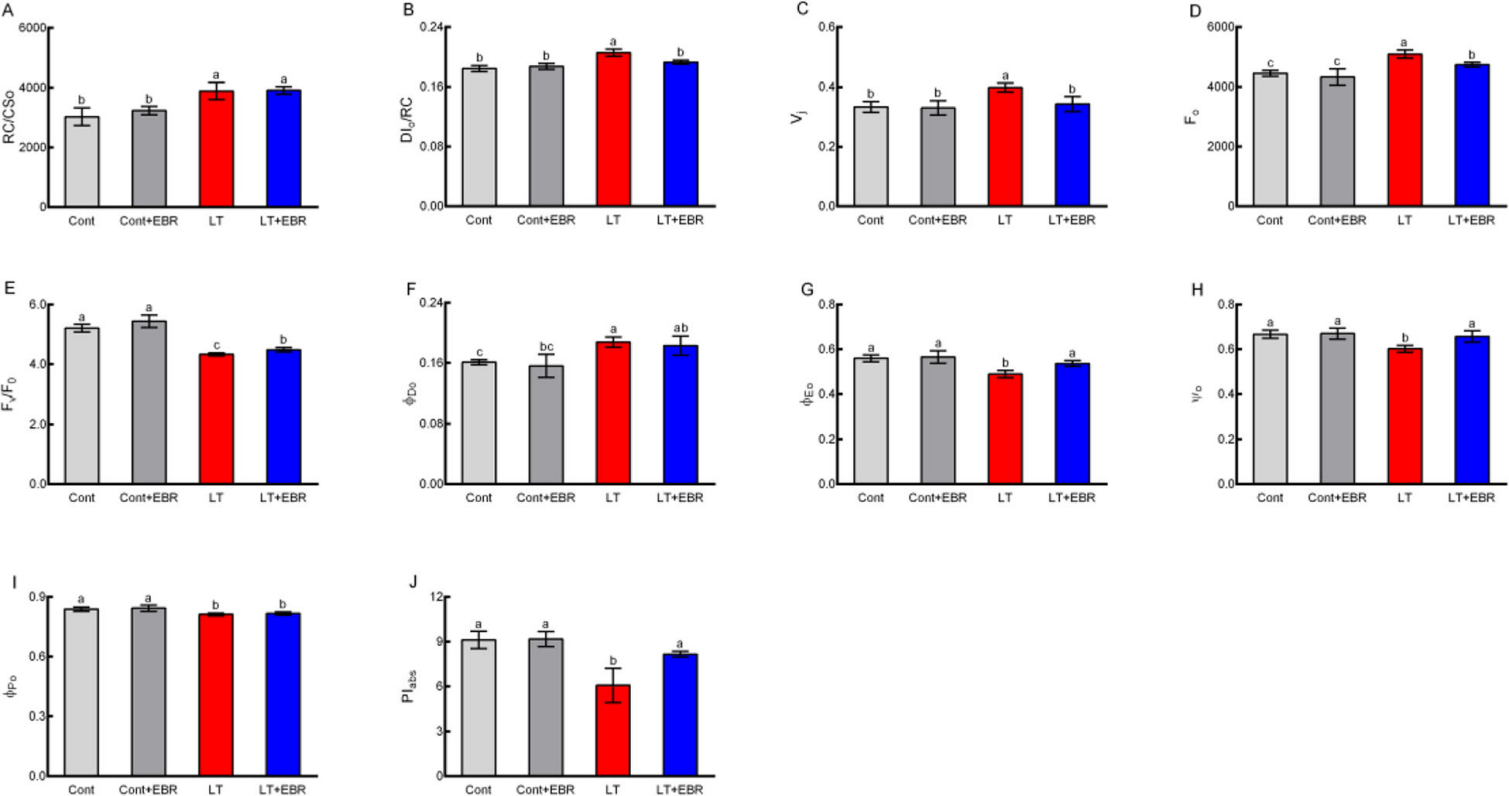
Effects of exogenous EBR application on chlorophyll fluorescence parameters in wucai leaves under LT stress. A-J represent RC/CS_0,_ DI_0_/RC, V_j_, F_0_, Fv/F_0_, φDo_,_ φEo_,_ ψ_o_, φPo and PI_abs_, respectively

The Chl *a* fluorescence transient reflects the effect of PSII afterquantitatively analyzing changes in the OJIP curve (Fig. 12). The radar maps show the changes in the receptor side and reflection center of PSII (Fig. 13). LT was associated with significantly higher V_j_, DI_0_/RC, and minimum fluorescence (F_0_) than the other 3 groups. In contrast, Fv/F_0_, φEo_,_ ψ_o_, and PI_abs_ in LT plants were significantly lower compared to the other 3 groups (Fig. 14, Additional file 12: Table S8). For all of the parameters, there were no significant differences detected between Cont and Cont+EBR. There were also no significant differences detected in RC/CS_0_, φDo_,_ and φPo between LT and LT + EBR. LT was associated with significantly higher V_j_, DI_0_/RC, and F_0_ compared to Cont, Cont+EBR, and LT + EBR. In contrast, Fv/F_0_, φEo, ψ_o_, and PI_abs_ in LT plants were significantly lower compared to the other 3 groups. There were no significant differences detected between Cont and Cont+EBR for all of the parameters. There were also no significant differences detected in RC/CS_0_, φDo, and φPo between LT and LT + EBR; however, a 3.18% decrease in φPo was observed when LT was compared to Cont. As for RC/CS_0_ and φDo, there were significant increases of 28.37 and 16.58%, respectively, in LT compared to Cont. In LT + EBR, the V_j_, DI_0_/RC, F_0_, Fv/F_0_, φEo, ψ_o_, and PI_abs_ concentrations significantly changed compared to LT. Among the above-mentioned factors, V_j_, DI_0_/RC, and F_0_ were significantly reduced by 13.80, 6.24, and 7.00%, respectively. Conversely, Fv/F_0_, φEo, ψ_o_, and PI_abs_ increased significantly by 3.42, 9.69, 9.12, and 34.38%, respectively, in LT + EBR compared to LT (Fig. 14).

## Discussion

BRs are a series of naturally present plant steroids associated with several critical physiological and cellular plant processes when experiencing a variety of environmental stressors [21]. BRs are a category of steroidal growth regulators that promote stress tolerance and crop yield [22, 23]. Although previous studies on BRs have demonstrated that the functions of exogenous BRs can mitigate the damage induced by abiotic stress [24, 25], the associated regulatory mechanisms, especially the underlying molecular mechanisms, have received little attention. In this study, through physiological and biochemical analyses, it was demonstrated that an EBR pretreatment improved the cold tolerance of wucai. Then, in order to focus on the EBR-mediated variety in gene expression, RNA-Seq was used to investigate the part of EBR that responded to LT at the global transcriptome level. To the best of our knowledge, this study is the first demonstration of the molecular mechanisms of wucai responses to EBR treatments under LT. In order to determine the effect of EBR on LT tolerance, the discussion is focused on contrasting the LT and LT + EBR treatment groups. Based on the GO and KEGG analyses, DEGs correlated with the application of exogenous EBR under LT were notably enriched in genes associated with photosynthesis and chlorophyll biosynthesis.

### Physiological parameters verify the role of exogenous EBR

Variations in leaf structure patterns represent the adaptation of many species [26]. Higher SLA helps with higher potential evaporation requirements and wider foliar display, capturing more light for use in constant biomass investment [27]. MDA content is a main indicator for evaluating the degree of membrane oxidation. Low MDA content leads to a higher tolerance of LT stress. Soluble sugars play a prominent, central role in plant structure and metabolism at the cellular and whole organism levels [28]. MSI can be used to assess the integrity of the plasma membrane [29]; the higher the MSI value, the more complete the plasma membrane. In this study, the results of SLA, MSI, total soluble sugar content, and MDA indicate that EBR can significantly alleviate injury to LT. In order to explore the underlying molecular mechanisms, transcriptome sequencing was performed on LT and LT + EBR plants.

### Exogenous EBR improves chlorophyll biosynthesis by promoting the conversion of intermediates

Chlorophyll is an important component in photosynthesis, as it plays a role in the transmission, distribution, and transformation of light energy [20]. Chlorophyll content is closely related to photosynthetic rate and organic matter accumulation [30]. Some studies have shown that exogenous application of EBR accelerates the increase of chlorophyll content in plants under stress [31–33]. Chlorophyll metabolism is an intricate pathway that involves the biosynthesis and degradation of chlorophyll. Chlorophyll biosynthesis is catalyzed by a diverse set of enzymes and can be restrained by abating the activity of all of these enzymes [34]. The results of this study indicate that exogenous EBR treatment can increase the content of photosynthetic pigments under LT stress and promote a spectrum of enzymatic reactions in chlorophyll biosynthesis.

ALA is the first precursor of chlorophyll biosynthesis. Therefore, ALA biosynthesis has a considerable effect on the rate of chlorophyll biosynthesis [20]. In this study, a significant increase in endogenous ALA was detected in LT + EBR plants compared to LT. Additionally, the conversion of ALA to chlorophyll includes many intermediates such as PBG, Proto IX, Mg-Proto IX, and Pchl. EBR application caused significant increases in the ALA and PBG contents, and significant decreases in Proto IX, Mg-Proto IX, and Pchl compared to LT. Four transcripts encoding 4 of the key enzymes involved in catalyzing the synthesis of these products were all up-regulated by exogenous EBR application. *CHLM* encodes a magnesium-protoporphyrin O-methyltransferase (EC:2.1.1.11), which catalyzes the transformation of Mg-protoporphyrin IX to Mg-protoporphyrin IX 13-monomethyl ester, accompanied by a significant decrease in Mg-Proto IX. *POR* encodes a protochlorophyllide reductase (EC:1.3.1.33), which catalyzes the transformation of protochlorophyllide to chlorophyllide a, accompanied by a significant decrease in Pchl and a significant increase in photosynthetic pigments. These results suggest that applications of exogenous EBR can modulate the synthesis of endogenous ALA and its transformation to intermediate products, thereby improving chlorophyll biosynthesis.

### Exogenous EBR weakened stress-induced photoinhibition and enhanced photosynthesis

Photosynthesis is among the primary plant processes that are affected most frequently by abiotic stress [35, 36]. PSII is believed to play an important role in plant responses to environmental stress [37]. For example, chilling stress has been shown to inhibit leaf photosynthesis through PSII-related photoinhibition [38, 39]. Chlorophyll fluorescence transients quantitatively explain dynamic changes in the OJIP curve, which in turn quantitatively explains the PSII light energy absorption, conversion, electron transport, PSII action center, activity on the receptor and donor sides, and redox state of the electron transporter. In this study, GO and KEGG enrichment analyses suggest that EBR-mediated induction of photosynthesis, chloroplast thylakoid membrane, and chlorophyll biosynthesis may be the primary factors contributing to the distinct photosynthetic characteristics of the EBR transcriptome under LT conditions.

In contrast, the PSII activity level, which is reflected by *ϕ*_Po_, was not affected in plants sprayed with EBR under normal temperature conditions (Fig. 7). *ϕ*_Po_ decreased in both LT and LT + EBR plants, especially in LT. This suggests that exogenous EBR can protect PSII under LT stress. Changes in F_0_ depend on the factors present during energy dissipation and PSII inactivation [40]. PSII damage or inactivation can result in an increase in F_0_ [41]. In this study, F_0_ increased under LT. However, the increase was smaller in LT + EBR than in LT (Fig. 7), indicating there was less relative damage to PSII in LT + EBR. Thus, exogenous EBR could alleviate damage caused by LT to photosynthetic reaction centers and reduce chilling photoinhibition.

The health of PSII is often used to determine the photochemical efficiency of the light reactions in photosynthesis II [42]. Compared to LT plants, the exogenous application of EBR in wucai promoted the maintenance of photochemical health under LT stress due to higher ΦPSII activity. From the KEGG pathway analysis, EBR application significantly regulated a series of proteins involved in PSII, including the oxygen-evolving enhancer, 10 kDa and Psb27 proteins. The direct effect of EBR on these PSII proteins revealed that EBR could bind to PSII membranes in order to maintain their integrity and improve photosynthetic function despite osmotic stress [17].

## Conclusion

This study provides insight into the function of exogenous EBR application on gene expression and predicted functions in wucai responding to LT stress. Compared to LT, SLA and MSI were significantly improved in LT + EBR. Additionally, the content of MDA and total soluble sugar significantly decreased in LT + EBR, indicating that exogenous EBR could alleviate LT injury. The transcriptome analysis revealed that EBR primarily affected the transcripts associated with photosynthesis, porphyrin and chlorophyll metabolism under LT stress. Specifically, a significant increase in chlorophyll content and significant changes in the chlorophyll fluorescence parameters (i.e., V_j_, Fv/F_0_, and PI_abs_) were detected when LT + EBR was compared to LT. The regulation of the transcriptome in combination with the physiology of chlorophyll biosynthesis and photosynthesis demonstrates the protective mechanism of EBR treatment in response to LT stress. The DEGs identified here could be further investigated as molecular markers, or used in future functional analyses investigating responses to LT stress and EBR application.

## Methods

### Plant material and stress treatment

Wucai seeds were provided by the Vegetable Genetics and Breeding Laboratory of the College of Horticulture, Anhui Agricultural University. Seeds were germinated from plugs before being transplanted into pots containing a mixture of stroma and vermiculite (3:1, *v/v*). Seedlings were grown in a growth chamber at 25/18 °C (day/night), 300 μmol m^− 2^ s^− 1^ photon flux density, and 70% relative humidity with a 12:12 h photoperiod.

In a pilot study, 6 levels of EBR concentrations (0, 0.05, 0.08, 0.1, 0.12, and 0.15 μM) were explored for treating wucai plants following a previously described protocol [21]. Based on the results of the pilot study, 0.1 μM EBR was identified as the optimum EBR concentration, which was subsequently used as the processing condition in the main experiment (Additional file 4: Figure S4).

Seedlings of uniform size at the 5–6 leaf stage (45 dafter planting) were selected and randomly divided into 2 groups for pretreatment. One group (*n* = 50) was sprayed with a 0.1 μM EBR solution, while the other group (*n* = 50) was sprayed with the same volume of ddH_2_O. Two d after pretreatment spraying, the seedlings in each pretreatment group were further divided into 2 groups. One group (*n* = 25) continued to grow in the suitable environment without LT stress, while the other group (*n* = 25) was grown under LT. In total, there were 4 treatment groups: (1) Cont plants grown in an ideal environment with the ddH_2_O pretreatment; (2) Cont+EBR plants grown in an ideal environment with the 0.1 μM EBR pretreatment; (3) LT plants grown with the ddH_2_O pretreatment; and (4) LT + EBR plants grown with the 0.1 μM EBR pretreatment. Seedlings in both the LT and LT + EBR groups were transferred to a phytotron set at 7 /3 °C (day/night), 300 μmol m^− 2^ s^− 1^ photon flux density, and 70% relative humidity with a 12:12 h photoperiod. Each treatment was replicated 3 times in the phytotron, and each treatment consisted of 25 plants. After 5 d of LT, functional leaf samples from 3 biological replicates were collected from LT and LT + EBR for sequencing. All of the samples were collected at the same time, ground into powder in liquid nitrogen, and immediately stored at − 80 °C for future analyses.

### Measurement the contents of SLA, MSI, Total soluble sugar and MDA

SLA was determined according to Choong et al. [43] with minor modifications. SLA was calculated by dividing the area of 10 diameter leaf discs (0.6 mm). Then, the leaf samples were placed in a constant temperature drying oven set at 105 °C for 15 min, and subsequently dried at 75 °C to a constant dry weight. SLA was calculated as the ratio of the leaf area to dry leaf weight.

MSI was determined according to methods previously described by Souhir et al. [44] with minor modifications. Briefly, the same quantity of leaves was placed in a plugged test tube containing 10 mL ddH_2_O. Then the tube was placed in a 40 °C water bath for 30 min. Afterwards, the conductivity was measured (C1). Then, the tube was transferred to a 100 °C water bath for 10 min, cooled to room temperature, and the conductivity was measured again (C2). MSI was calculated using the following formula:

$$ \mathrm{MSI}=\left[1-\left(\mathrm{C}1\div \mathrm{C}2\right)\times 100\%\right] $$

After grinding, dry leaf samples were mixed with 4 mL 80% alcohol and shaken at 80 °C for 30 min. Cooled samples were centrifuged at 6855 r/min for 3 min, and the supernatant was collected. The residue was washed with 80% alcohol 3 times, and the supernatant was extracted after each wash. Then, all of the supernatants were brought to a set volume of 10 mL with 80% alcohol and decolorized with 0.01 g activated carbon at 80 °C for 30 min. After the addition of activated carbon, the material was filtered to obtain a filtrate, which was mixed with 6 mL anthrone and heated in a boiling water bath for 10 min. Samples were then ice-cooled and kept at room temperature (25 °C) for 10 min. After the chromogenic reaction, the optical density (OD) was spectrophotometrically recorded at 620 nm.

MDA is a product of lipid peroxidation, associated with plant oxidative stress. The MDA level of all of the plant samples was assessed through lipid peroxidation following the methods previously described by Mohammadi et al. [45] with minor modifications. Fresh leaf samples (0.5 g) were homogenized in 10 mL trichloroacetic acid (10%) and centrifuged at 4000 r/min for 10 min. Then, 2.0 mL supernatant was mixed with 2 mL thiobarbituric acid (0.6%), heated at 100 °C for 15 min, and cooled to room temperature. The absorbance of each aliquot was measured at 450 (A_450_), 532 (A_532_), and 600 nm (A_600_). The MDA content was calculated using the following formula:

$$ \mathrm{MDA}=6.45\times \left({\mathrm{A}}_{532}\hbox{-} {\mathrm{A}}_{600}\right)-0.56\times {\mathrm{A}}_{450}. $$

### RNA extraction, library construction and RNA sequencing

Total RNA was extracted from the LT and LT + EBR leaf samples using a mirVana miRNA Isolation Kit (Ambion, TX, USA) following the manufacturer’s instructions. RNA integrity was evaluated using an Agilent 2100 Bioanalyzer (Agilent Technologies, CA, USA). Samples with an RNA Integrity Number (RIN) ≥ 7 were subjected to subsequent analysis. Libraries were constructed using the TruSeq Stranded mRNA LT Sample Prep Kit (Illumina, CA, USA) following the manufacturer’s instructions. Then these libraries were sequenced on the HiSeqTM 2500 or HiSeq X Ten Illumina sequencing platform (Illumina, CA, USA), which generated 125 bp/150 bp paired-end reads.

### Sequence assembly and annotation

Each library generated > 6 gigabytes of raw data. Clean reads were filtered from the raw sequencing data and low-quality reads containing unknown nucleotides or adaptor sequences were removed; this procedure was performed following the methods previously described by Bolger et al. [46]. Clean, filtered reads were aligned to the *B. rapa* reference genome by hisat2 [47].

### Differential expression analysis

A differential expression analysis was performed for both LT and LT + EBR treatments based on the DESeq R package [48], which allowed for statistical analysis using a negative binomial (NB) distribution model. To control the false discovery rate (FDR), the FDR value calculation method was used to calculate the *p*-value of each gene. Then, the FDR error control method was used to perform multi-hypothesis test corrections on the *p*-value, where an adjusted *p*-value < 0.05 and |log_2_(foldchange)| > 1 was accepted to represent DEGs.

GO enrichment analysis of the DEGs was performed according to the GOseq R package. GO terms with the number of DEGs > 2 were regarded as significantly enriched (*p* < 0.05). Statistical enrichment of the DEGs was carried out in KEGG pathways using KOBAS software [49].

### Validation of DEGs by qRT-PCR

Total RNA was extracted using the RNeasy extraction tool then reverse-transcribed into cDNA for the next analysis. A total of 20 transcripts were selected to verify the RNA-Seq analysis. *Actin* (*ACT*) was used as a reference gene to normalize the data. The SYBR fluorescent reagent (TaKaRa, RR820A) and Bio-Rad CFX96™ Real-Time System (Bio-Rad, CA, USA) were used to conduct qRT-PCR following the manufacturer’s instructions. The gene expression data were analyzed using the 2^−ΔΔCt^ relative quantitative methods [50] in Microsoft Excel.

### Measurement of photosynthetic pigment content and chlorophyll biosynthesis intermediate contents

Chlorophyll contents were determined using the methods previously described by Lichtenthaler et al. and Wellburn et al. [51] with minor modifications. Briefly, chlorophyll was extracted from 0.2 g leaves with 25 mL extracting solution (acetone/V: ethanol/V: water/V = 4.5: 4.5:1). The absorbance of the supernatant was measured at 3 wavelengths: 645, 652, and 663 nm. The contents of Chl *a*, Chl *b* and Total Chl were calculated using the following formulas:

$$ {\mathrm{C}}_{\mathrm{C}\mathrm{hla}}=13.95{\mathrm{A}}_{665}-6.88{\mathrm{A}}_{649}, $$

$$ {\mathrm{C}}_{\mathrm{C}\mathrm{hlb}}=24.96{\mathrm{A}}_{649}-7.32{\mathrm{A}}_{665}, $$

$$ \mathrm{Total}\ \mathrm{Chl}=\mathrm{Chl}\ \mathrm{a}+\mathrm{Chl}\ \mathrm{b} $$

In order to estimate the concentration of ALA, 0.5 g leaf samples were homogenized in 5 mL trichloroacetic acid (4%) and the extract was centrifuged at 14,541 r/min for 15 min. Then, 3 mL supernatant was added to 1.7 mL sodium acetate and 0.1 mL of acetylacetone. The mixture was placed in a boiling water bath for 10 min then cooled to room temperature. Two mL supernatant was thoroughly mixed with an Ehrlich-Hg reagent (0.2 g HgCl_2_ + 1 g P-dimethyl amino benzaldehyde + 42 mL glacial acetic acid + 8 mL 70% perchloric acid). After 15 min in darkness, the absorbance of the resulting solution was determined spectrophotometrically at 553 nm [52].

PBG content was measured following the methods previously described by Bogorad et al. [53]. Briefly, 0.5 g leaves were homogenized in 5 mL extraction buffer (0.6 M Tris and 0.1 M EDTA, pH was adjusted to 8.2 with HCl) and centrifuged for 10 min at 14,541 r/min. Two mL supernatant was thoroughly mixed with 2 mL Ehrlich-Hg reagent. After incubating in darkness for 15 min, the absorbance was measured at 553 nm.

The (Proto IX, Mg-Proto IX and Pchl contents were quantified following the methods previously described by Hodgins and Huystee et al. [54]. Briefly, leaf tissues were extracted with 80% alkaline acetone and centrifuged at 11,873 r/min for 10 min at 4 °C. The absorbance of the supernatant was measured at 575, 590 and 628 nm. The contents of Proto IX, Mg-Proto IX and Pchl were calculated using the formula previously described by Hodgins et al. [54].

### Measurement of chlorophyll *a* fluorescence transients

In vivo chlorophyll *a* fluorescence emissions were measured in 30 min dark-adapted leaves with a continuous excitation fluorometer Pocket Plant Efficiency Analyzer (PEA, Hansatech, UK). Data were sampled at 10 μs intervals for the first 300 μs, providing excellent time resolution of F_0_ and the initial rise kinetics. The time resolution of digitization was switched to slower acquisition rates as the kinetics of the fluorescence signal slowed. Chlorophyll *a* fluorescence transient was analyzed using the JIP-test formulae [55]. The fluorescence intensity at 20 μs was considered to be F_0_, while the maximal fluorescence intensity was presumed to be equal to F_m_, as the intensity was high enough to ensure the closure of all of the reaction centers (RCs) of PSII. Moreover, the fluorescence intensity at 300 μs (F300 μs), 2 ms (J-step, FJ), and 30 ms (I-step, FI) were also measured [56]. The following parameters refer to time 0 (the start of fluorescence induction): (a) the specific energy fluxes for absorption per reaction center (ABS/RC), trapping (TRo/RC), electron transport (ETo/RC), and dissipation at the level of the antenna chlorophylls (DIo/RC); and (b) the normalized total complementary area above the OJIP transient or total electron carriers per RC (Sm = EC_0_/RC = Area/(Fm-F_0_)). Performance index (PI) on absorption basis was calculated as: PI (abs) = (RC/ABS) • [φPo / (1– φPo)] [ψo / (1– ψo)]. Maximum quantum yield of primary photochemistry was calculated as: φPo = Fv / Fm = (Fm - Fo) / Fm [57]. Other parameters measured in this study are provided (Additional file 12: Table S8).

### Statistical analyses

All of the data from the 4 treatments were subjected to an analysis of variance (ANOVA). The mean separation was performed using the Fisher’s protected least significant difference (LSD) test with a significance level of *p* < 0.05. Analyses were conducted using SPSS v19.0 for Windows (SPSS Inc., USA). Figures were plotted using GraphPad Prism v7.0 and Origin Pro v9.1 software (OriginLab Corporation, MA, USA).

## Supplementary information

**Additional file 1: Figure S1.** The quality control of six samples. A-F represent LT-1, LT-2, LT-3, LT + EBR-1, LT + EBR-2, and LT + EBR-3, respectively.

**Additional file 2: Figure S2.** The reads mapping of six samples. A-F represent LT-1, LT-2, LT-3, LT + EBR-1, LT + EBR-2, and LT + EBR-3, respectively.

**Additional file 3: Figure S3.** The 20 DEGs we randomly selected for qRT-PCR assay in EBR-mediated LT stress.

**Additional file 4: Figure S4.** The results of identifying EBR concentration.

**Additional file 5: Table S1.** Summary of sequence assembly after illumine sequencing.

**Additional file 6: Table S2.** Number of reads sequenced and mapped to the *Brassica rapa* genome.

**Additional file 7: Table S3.** The expression patterns of the cold acclimation and cold-induced genes.

**Additional file 8: Table S4.** The expression patterns of the BR-responsive genes.

**Additional file 9: Table S5.** Primers of verifying genes.

**Additional file 10: Table S6.** Primers of photosynthesis genes.

**Additional file 11: Table S7.** DEGs of photosynthesis–antenna proteins and photosynthesis.

**Additional file 12: Table S8.** Parameters derived from the OJIP transient for use in the current study.

## Data Availability

The raw RNA-Seq data used in this study have been deposited in the Nation Center for Biotechnology Information (NCBI) Sequence Read Archive (SRA) database under the accession number SRP200451 (https://www.ncbi.nlm.nih.gov/sra/SRP200451).

## Abbreviations

ALA: 5-aminolevulinic acid
*B. rapa*: *Brassica rapa*
BR: Brassinosteroids
Chl *a*: Chlorophyll *a*
Chl *b*: Chlorophyll *b*
Cont: Control
Cont+EBR: Control with 24-epibrassinolide
DEG: Different expression gene
EBR: 24-epibrassinolide
GO: Gene Ontology
KEGG: Kyoto Encyclopedia of Genes and Genomes
LT + EBR: Low temperature with 24-epibrassinolide
LT: Low temperature
MDA: Malondialdehyde
Mg-proto IX: Mg-protoporphyrin IX
MSI: Membrane stability index
PBG: Porphobilinogen
Pchl: Protochlorophyllide
Proto IX: Protoporphyrin IX
PSI: Photosystem I
PSII: Photosystem II
qRT-PCR: Real time quantitative PCR
RPKMs: The reads per kilo bases per million reads
SLA: Specific leaf area
Total Chl: Total Chlorophyll
