# Supplementary material for: Transcriptome analysis reveals a positive effect of brassinosteroids on the photosynthetic capacity of wucai under low temperature
Source: BMC Genomics. 2019 Nov 6;20:810. doi: 10.1186/s12864-019-6191-2 (PMC6836548; doi:10.1186/s12864-019-6191-2)
Supplement: Supplementary file 1 — Additional file 1: Figure S1. The quality control of six samples. A-F represent LT-1, LT-2, LT-3, LT + EBR-1, LT + EBR-2, and LT + EBR-3, respectively. [file 12864_2019_6191_MOESM1_ESM.docx]

**Fig. S1 The quality control of six samples.** A-F represent LT-1_,_ LT-2, LT-3, LT+EBR-1, LT+EBR-2, and LT+EBR-3, respectively.

F

E

D


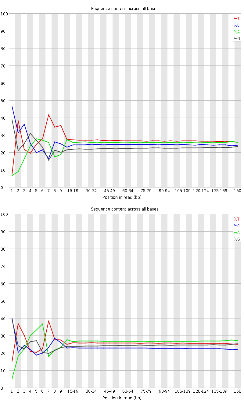

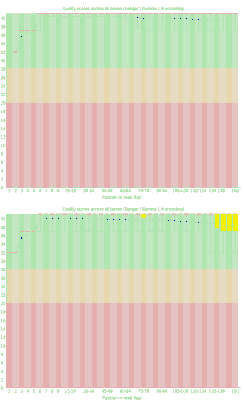

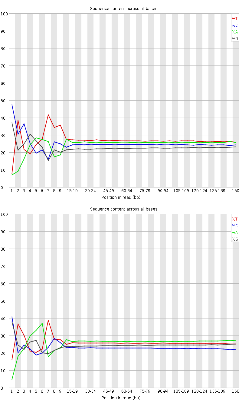

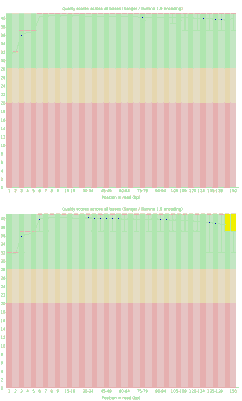

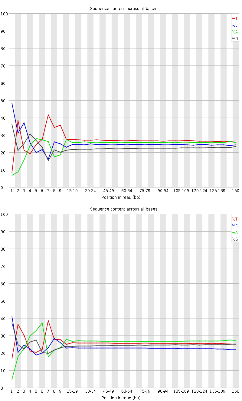

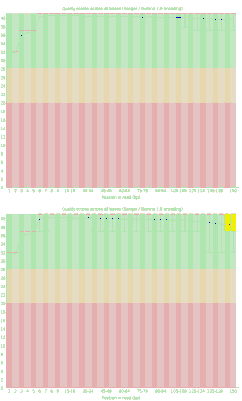

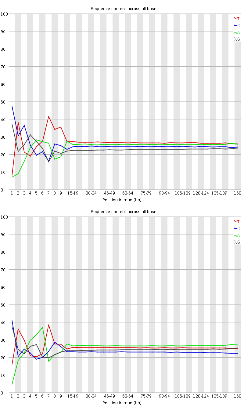

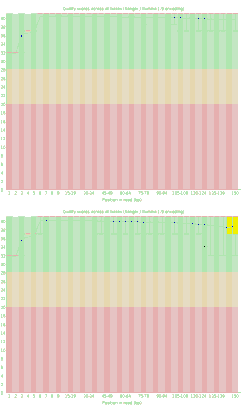

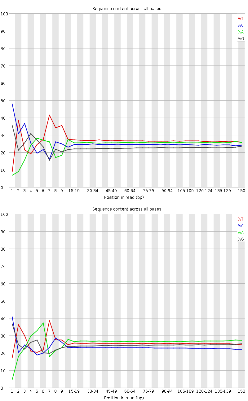

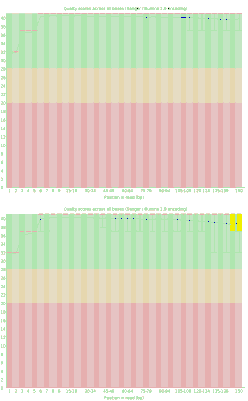

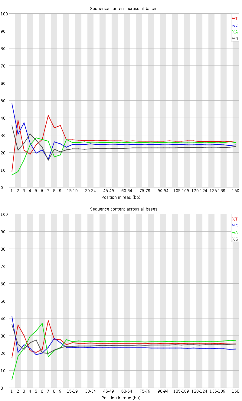

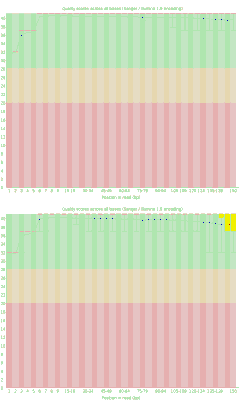


A

B

C
