# Supplementary figures and images for: Transcriptome analysis reveals a positive effect of brassinosteroids on the photosynthetic capacity of wucai under low temperature
Source: BMC Genomics. 2019 Nov 6;20:810. doi: 10.1186/s12864-019-6191-2 (PMC6836548; doi:10.1186/s12864-019-6191-2)

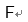

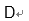

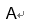

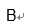

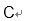

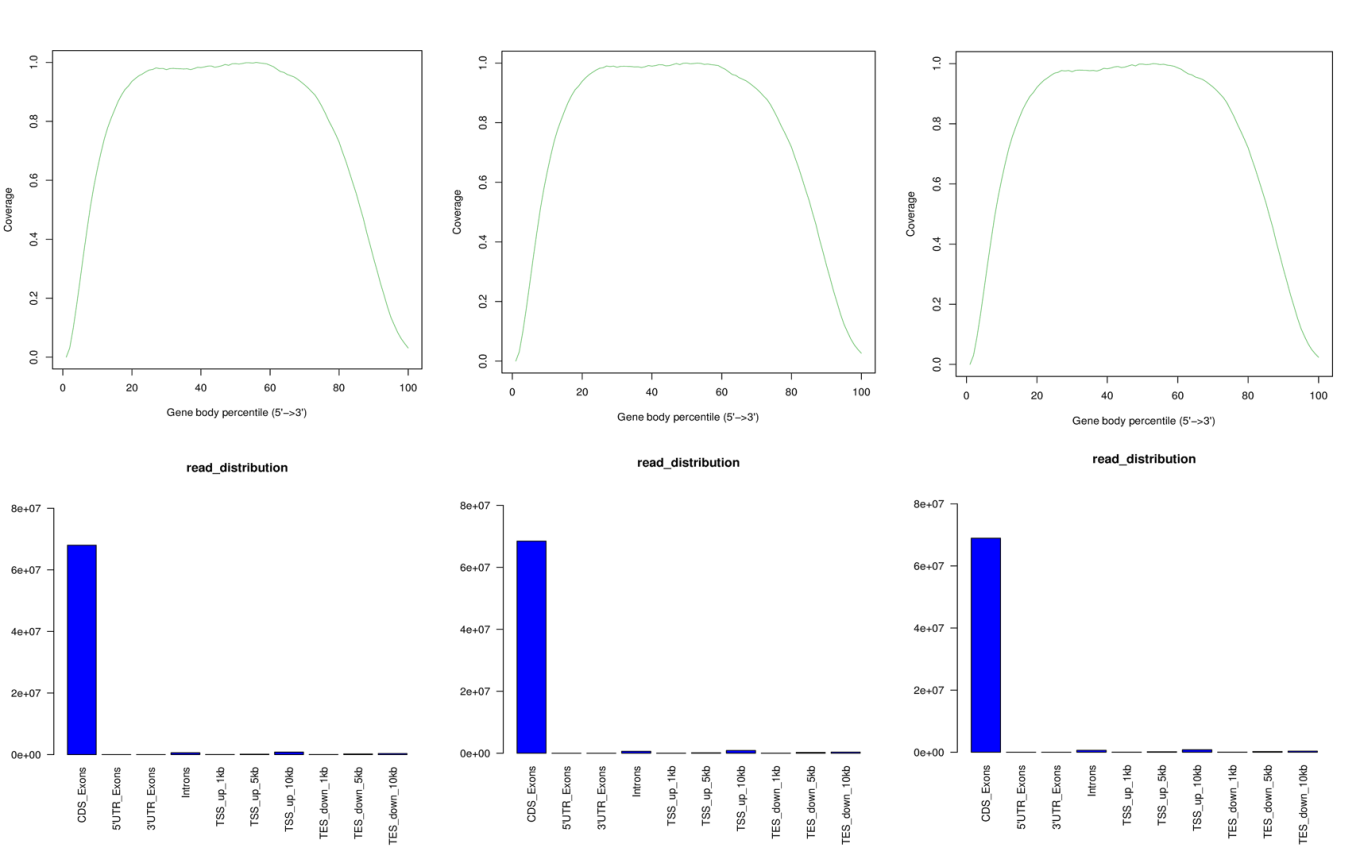


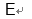

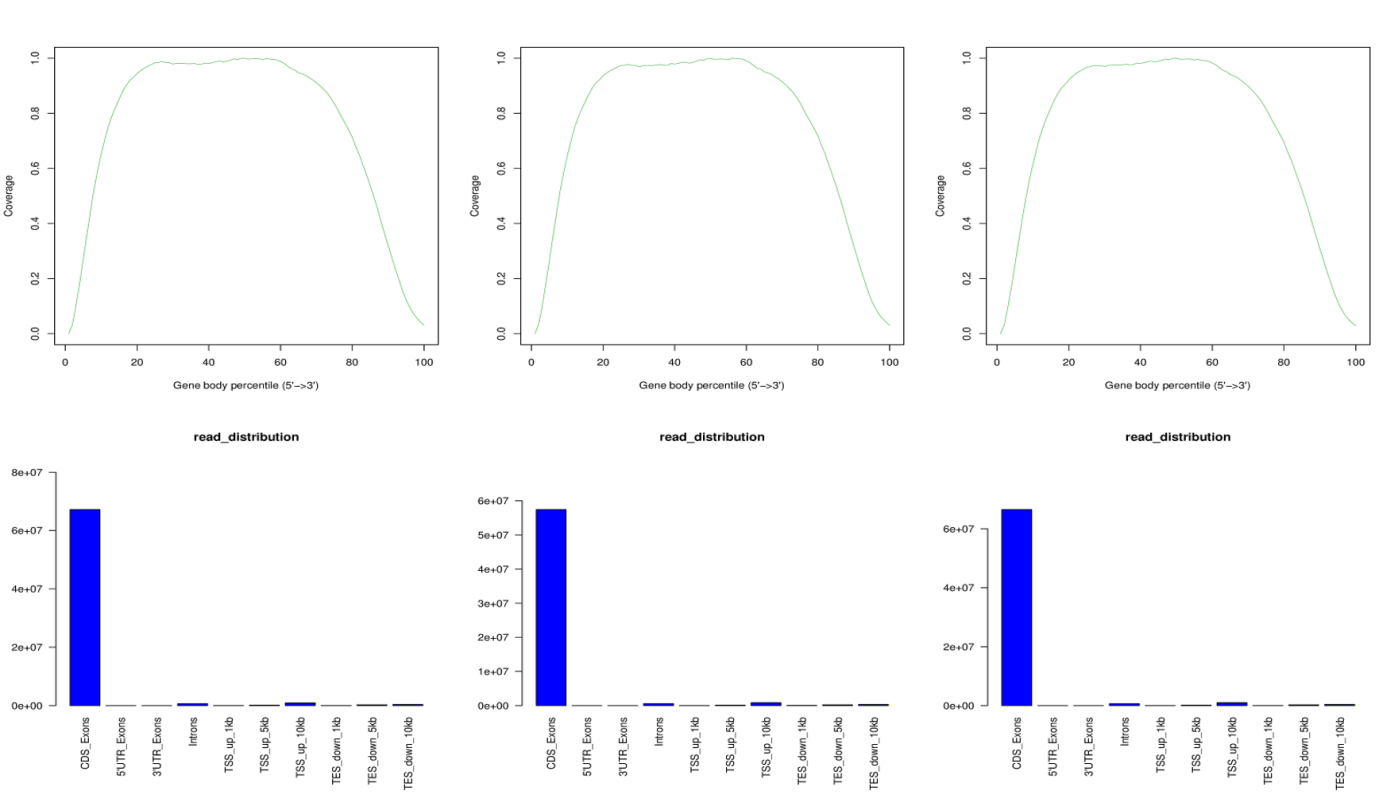


**Fig. S2 The reads mapping of six samples.** A-F represent LT-1, LT-2, LT-3, LT+EBR-1, LT+EBR-2, and LT+EBR-3, respectively.

Supplement: Supplementary file 2 — Additional file 2: Figure S2. The reads mapping of six samples. A-F represent LT-1, LT-2, LT-3, LT + EBR-1, LT + EBR-2, and LT + EBR-3, respectively. [file 12864_2019_6191_MOESM2_ESM.docx]
