# Supplementary material for: Transcriptome analysis reveals a positive effect of brassinosteroids on the photosynthetic capacity of wucai under low temperature
Source: BMC Genomics. 2019 Nov 6;20:810. doi: 10.1186/s12864-019-6191-2 (PMC6836548; doi:10.1186/s12864-019-6191-2)
Supplement: Supplementary file 3 — Additional file 3: Figure S3. The 20 DEGs we randomly selected for qRT-PCR assay in EBR-mediated LT stress. [file 12864_2019_6191_MOESM3_ESM.docx]

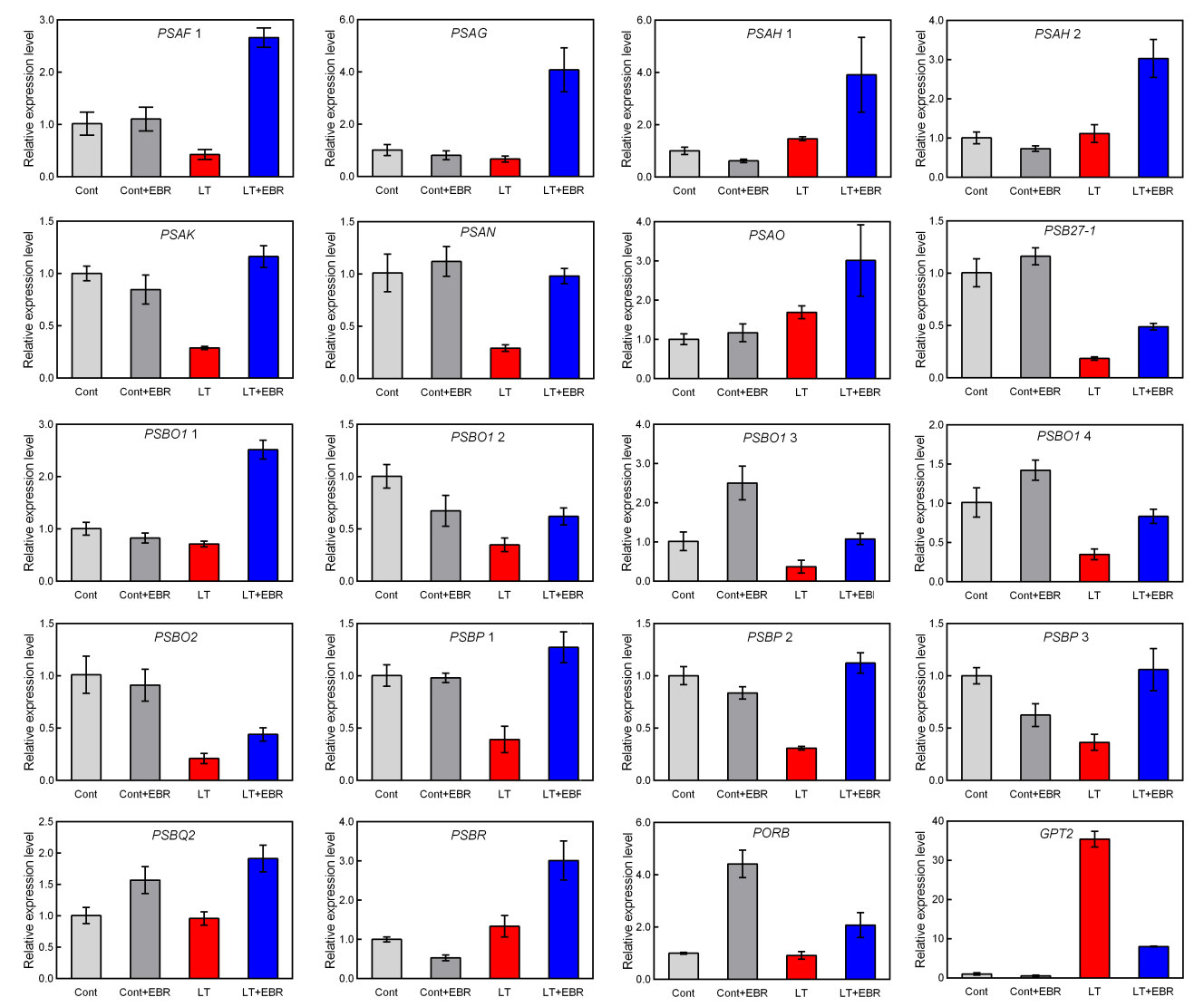


**Fig. S3 The 20 DEGs we randomly selected for qRT-PCR assay in EBR-mediated LT stress.** Cont, suitable environment control plants without 0.1 µM EBR pretreatment; Cont+EBR, suitable environment plants with 0.1 µM EBR pretreatment; LT, low temperature control plants without 0.1 µM EBR pretreatment; LT+EBR, low temperature-stressed plants with 0.1 µM EBR pretreatment.
