# Supplementary material for: Transcriptome analysis reveals a positive effect of brassinosteroids on the photosynthetic capacity of wucai under low temperature
Source: BMC Genomics. 2019 Nov 6;20:810. doi: 10.1186/s12864-019-6191-2 (PMC6836548; doi:10.1186/s12864-019-6191-2)
Supplement: Supplementary file 4 — Additional file 4: Figure S4. The results of identifying EBR concentration. [file 12864_2019_6191_MOESM4_ESM.docx]

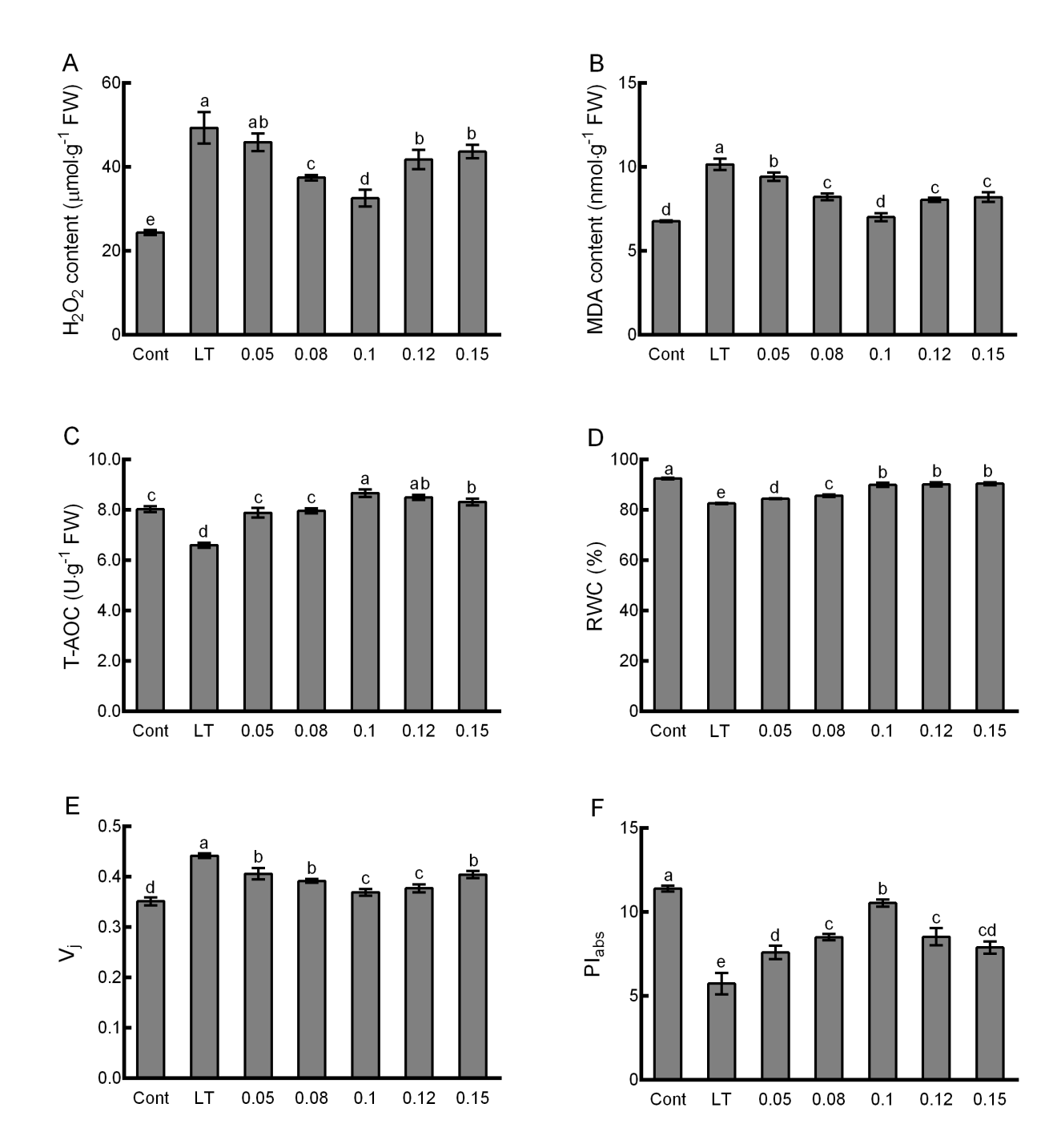


**Fig. S4 The results of identifying** **EBR concentration.** Cont, suitable environment control plants without EBR pretreatment; LT, low temperature control plants without EBR pretreatment; 0.05, low temperature-stressed plants with 0.05 µM EBR pretreatment; 0.08, low temperature-stressed plants with 0.08 µM EBR pretreatment; 0.1, low temperature-stressed plants with 0.1 µM EBR pretreatment; 0.12, low temperature-stressed plants with 0.12 µM EBR pretreatment; 0.15, low temperature-stressed plants with 0.15 µM EBR pretreatment.
