# Supplementary material for: Transcriptome analysis reveals a positive effect of brassinosteroids on the photosynthetic capacity of wucai under low temperature
Source: BMC Genomics. 2019 Nov 6;20:810. doi: 10.1186/s12864-019-6191-2 (PMC6836548; doi:10.1186/s12864-019-6191-2)
Supplement: Supplementary file 5 — Additional file 5: Table S1. Summary of sequence assembly after illumine sequencing. [file 12864_2019_6191_MOESM5_ESM.docx]

Table S1

Summary of sequence assembly after illumine sequencing.

| Sample name^a^ | Raw reads | | Clean reads | | Clean bases | | Valid bases (%) | | Q30^b^ (%) | | GC content^c^ (%) | |  |
| --- | --- | --- | --- | --- | --- | --- | --- | --- | --- | --- | --- | --- | --- |
| LT-1 | | 53.49M | | 51.73M | | 7.33G | | 91.40 | | 94.46 | | 47.44 | |
| LT-2 | | 53.28M | | 51.81M | | 7.39G | | 92.45 | | 95.20 | | 47.49 | |
| LT-3 | | 53.76M | | 52.16M | | 7.44G | | 92.24 | | 95.10 | | 47.48 | |
| LT+EBR-1 | | 54.20M | | 52.31M | | 7.37G | | 90.67 | | 94.68 | | 47.73 | |
| LT+EBR-2 | | 45.91M | | 44.62M | | 6.30G | | 91.44 | | 95.26 | | 47.67 | |
| LT+EBR-3 | | 52.37M | | 50.78M | | 7.25G | | 92.30 | | 94.96 | | 47.70 | |

^a^ The numbers 1,2 and 3 at the end of the sample name represent three replicates, respectively. LT-samples treated with water; LT+EBR-samples treated with 0.1 μM EBR.

^b^ Percentage of bases with a Phred value of at least 30.

^c^ Proportion of guanidine and cytosine nucleotides among total nucleotides.
