# Supplementary material for: Transcriptome analysis reveals a positive effect of brassinosteroids on the photosynthetic capacity of wucai under low temperature
Source: BMC Genomics. 2019 Nov 6;20:810. doi: 10.1186/s12864-019-6191-2 (PMC6836548; doi:10.1186/s12864-019-6191-2)
Supplement: Supplementary file 6 — Additional file 6: Table S2. Number of reads sequenced and mapped to the Brassica rapa genome. [file 12864_2019_6191_MOESM6_ESM.docx]

Table S2

Number of reads sequenced and mapped to the Brassica rapa genome.

| Sample name | LT-1 | LT-2 | LT-3 | LT+EBR-1 | LT+EBR-2 | LT+EBR-3 |
| --- | --- | --- | --- | --- | --- | --- |
| Total reads | 51734312 | 51814486 | 52155790 | 52313928 | 44619828 | 50780574 |
| Total Mapped | 45181216  (87.33%) | 45347257  (87.52%) | 45624864  (87.48%) | 45340591  (86.67%) | 38716012  (86.77%) | 44462789  (87.56%) |
| Multiple mapped | 1250813  (2.42%) | 1251419  (2.42%) | 1232323  (2.36%) | 1280178  (2.45%) | 1066916  (2.39%) | 1263612  (2.49%) |
| Uniquely mapped | 43930403  (84.92%) | 44095838  (85.10%) | 44392541  (85.12%) | 44060413  (84.22%) | 37649096  (84.38%) | 43199177  (85.07%) |
| Read-1 | 22010028  (42.54%) | 22081446  (42.62%) | 22233805  (42.63%) | 22060057  (42.17%) | 18855327  (42.26%) | 21638667  (42.61%) |
| Read-2 | 21920375  (42.37%) | 22014392  (42.49%) | 22158736  (42.49%) | 22000356  (42.05%) | 18793769  (42.12%) | 21560510  (42.46%) |
| Reads map to '+' | 21983714  (42.49%) | 22067123  (42.59%) | 22215890  (42.60%) | 22056148  (42.16%) | 18846280  (42.24%) | 21621499  (42.58%) |
| Reads map to '-' | 21946689  (42.42%) | 22028715  (42.51%) | 22176651  (42.52%) | 22004265  (42.06%) | 18802816  (42.14%) | 21577678  (42.49%) |
| Non-splice reads | 24762411  (47.86%) | 24717860  (47.70%) | 24923921  (47.79%) | 25413147  (48.58%) | 21621228  (48.46%) | 24466935  (48.18%) |
| Splice reads | 19167992  (37.05%) | 19377978  (37.40%) | 19468620  (37.33%) | 18647266  (35.64%) | 16027868  (35.92%) | 18732242  (36.89%) |
