# Supplementary material for: Transcriptome analysis reveals a positive effect of brassinosteroids on the photosynthetic capacity of wucai under low temperature
Source: BMC Genomics. 2019 Nov 6;20:810. doi: 10.1186/s12864-019-6191-2 (PMC6836548; doi:10.1186/s12864-019-6191-2)
Supplement: Supplementary file 7 — Additional file 7: Table S3. The expression patterns of the cold acclimation and cold-induced genes. [file 12864_2019_6191_MOESM7_ESM.docx]

Table S3

The expression patterns of the cold acclimation and cold-induced genes.

| Gene_ ID | *P* val | Up  Down | Description | Synonym | GO_ term |
| --- | --- | --- | --- | --- | --- |
| LOC103827659 | 6.27E-22 | Up | Ethylene-responsive transcription factor 5 | ERF5 | response to cold |
| LOC103827754 | 0.00298 | Up | Zinc finger protein AZF1 | AZF1 | response to cold |
| LOC103829997 | 1.37E-28 | Up | Chlorophyll a-b binding protein 6, chloroplastic | LHCA1 | response to cold |
| LOC103837002 | 8.39E-89 | Up | Chlorophyll a-b binding protein 2.4, chloroplastic | LHCB2.4 | response to cold |
| LOC103838389 | 0.001343 | Up | Lipid transfer protein EARLI 1 | EARLI1 | response to cold |
| LOC103838443 | 3.19E-69 | Up | Photosystem I chlorophyll a/b-binding protein 3-1, chloroplastic | LHCA3 | response to cold |
| LOC103839241 | 0.008651 | Up | RNA-binding protein CP29B, chloroplastic | CP29B | response to cold |
| LOC103839886 | 3.65E-25 | Up | Lipid transfer protein EARLI 1 | EARLI1 | response to cold |
| LOC103840296 | 7.37E-07 | Up | RNA-binding protein CP29B, chloroplastic | CP29B | response to cold |
| LOC103840935 | 3.73E-05 | Up | Ethylene-responsive transcription factor ERF011 | ERF011 | response to cold |
| LOC103841392 | 4.10E-31 | Up | Chlorophyll a-b binding protein 6, chloroplastic | LHCA1 | response to cold |
| LOC103843334 | 9.50E-05 | Up | U2 small nuclear ribonucleoprotein A' | At1g09760 | response to cold |
| LOC103843995 | 1.79E-16 | Up | Cellulose synthase-like protein D3 | CSLD3 | response to cold |
| LOC103844559 | 0.009743 | Up | Protein SENESCENCE-ASSOCIATED GENE 21, mitochondrial | SAG21 | response to cold |
| LOC103848198 | 0.001556 | Up | Lipid transfer protein EARLI 1 | EARLI1 | response to cold |
| LOC103851359 | 5.61E-05 | Up | Germin-like protein subfamily 3 member 3 | GER3 | response to cold |
| LOC103857735 | 8.48E-06 | Up | Aldo-keto reductase family 4 member C9 | AKR4C9 | response to cold |
| LOC103858633 | 1.47E-22 | Up | Lipid transfer protein EARLI 1 | EARLI1 | response to cold |
| LOC103859119 | 0.000347 | Up | Hydrophobic protein RCI2A | RCI2A | response to cold |
| LOC103860327 | 0.005913 | Up | Chlorophyll a-b binding protein 2.1, chloroplastic | LHCB2.1 | response to cold |
| LOC103862822 | 3.05E-59 | Up | Photosystem I chlorophyll a/b-binding protein 2, chloroplastic | LHCA2 | response to cold |
| LOC103870579 | 5.03E-09 | Up | Rhodanese-like domain-containing protein 10 | STR10 | response to cold |
| LOC103870985 | 6.02E-24 | Up | Beta carbonic anhydrase 1, chloroplastic | BCA1 | response to cold |
| LOC103873241 | 1.22E-89 | Up | Chlorophyll a-b binding protein 4, chloroplastic | LHCA4 | response to cold |
| LOC103874060 | 0.01175 | Up | VIN3-like protein 2 | VIL2 | response to cold |
| LOC103875194 | 2.57E-127 | Up | Chlorophyll a-b binding protein 2.4, chloroplastic | LHCB2.4 | response to cold |
| LOC103249160 | 0.001488 | Up | Zinc finger protein ZAT12 | ZAT12 | cold acclimation |
| LOC103830030 | 0.000445 | Up | Protein ESKIMO 1 | ESK1 | response to freezing |
| LOC103835009 | 3.39E-24 | Up | Cold-regulated 413 plasma membrane protein 4 | At4g37220 | integral component of membrane |
| LOC103837653 | 0.000991 | Up | pEARLI1-like lipid transfer protein 1 | AZI1 | cold acclimation |
| LOC103844161 | 1.02E-11 | Up | Glutamate receptor 3.4 | GLR3.4 | cellular response to cold |
| LOC103845908 | 6.27E-07 | Up | Cold shock domain-containing protein 3 | CSP3 | cold acclimation |
| LOC103828680 | 3.32E-16 | Down | Probable mediator of RNA polymerase II transcription subunit 37e | MED37E | response to cold |
| LOC103832452 | 0.018208 | Down | Cinnamoyl-CoA reductase 2 | CCR2 | response to cold |
| LOC103832872 | 0.043814 | Down | Hexokinase-3 | At1g50460 | response to cold |
| LOC103833106 | 7.86E-21 | Down | Photosystem I chlorophyll a/b-binding protein 5, chloroplastic | LHCA5 | response to cold |
| LOC103834289 | 4.92E-50 | Down | Early light-induced protein 1, chloroplastic | ELIP1 | response to cold |
| LOC103836185 | 4.47E-06 | Down | Lipid transfer protein EARLI 1 | EARLI1 | response to cold |
| LOC103841629 | 0.000189 | Down | Glucan endo-1,3-beta-glucosidase, acidic isoform | BG2 | response to cold |
| LOC103842320 | 0.022902 | Down | Glucan endo-1,3-beta-glucosidase, acidic isoform | BG2 | response to cold |
| LOC103845911 | 3.72E-05 | Down | Protein EARLY-RESPONSIVE TO DEHYDRATION 7, chloroplastic | ERD7 | response to cold |
| LOC103850006 | 7.89E-34 | Down | Hydrophobic protein RCI2B | RCI2B | response to cold |
| LOC103856668 | 0.003038 | Down | Phosphoinositide phospholipase C 1 | PLC1 | response to cold |
| LOC103857811 | 6.93E-10 | Down | Annexin D4 | ANN4 | response to cold |
| LOC103857812 | 8.17E-05 | Down | Annexin D3 | ANN3 | response to cold |
| LOC103859694 | 0.016777 | Down | Phytochrome B | PHYB | response to cold |
| LOC103859976 | 4.48E-16 | Down | Early light-induced protein 1, chloroplastic | ELIP1 | response to cold |
| LOC103861574 | 0.004912 | Down | Low-temperature-induced 65 kDa protein | LTI65 | response to cold |
| LOC103862449 | 0.008098 | Down | Senescence/dehydration-associated protein At4g35985, chloroplastic | P85 | response to cold |
| LOC103865686 | 0.001109 | Down | Annexin D3 | ANN3 | response to cold |
| LOC103868622 | 0.00108 | Down | Hexokinase-3 | At1g50460 | response to cold |
| LOC103868967 | 2.38E-34 | Down | Early light-induced protein 1, chloroplastic | ELIP1 | response to cold |
| LOC103873927 | 4.55E-15 | Down | Annexin D2 | ANN2 | response to cold |
| LOC103874051 | 0.001535 | Down | Zinc finger protein AZF1 | AZF1 | response to cold |
| LOC103857283 | 0.000299 | Down | Dehydration-responsive element-binding protein 1B | DREB1B | cold acclimation |
| LOC103845911 | 3.72E-05 | Down | Protein EARLY-RESPONSIVE TO DEHYDRATION 7, chloroplastic | ERD7 | response to cold |
