# Supplementary material for: Transcriptome analysis reveals a positive effect of brassinosteroids on the photosynthetic capacity of wucai under low temperature
Source: BMC Genomics. 2019 Nov 6;20:810. doi: 10.1186/s12864-019-6191-2 (PMC6836548; doi:10.1186/s12864-019-6191-2)
Supplement: Supplementary file 8 — Additional file 8: Table S4. The expression patterns of the BR-responsive genes. [file 12864_2019_6191_MOESM8_ESM.docx]

Table S4

The expression patterns of the BR-responsive genes.

| Gene_ ID | *P* val | Up  Down | Description | Synonym | GO_ term |
| --- | --- | --- | --- | --- | --- |
| LOC103828402 | 3.40E-12 | Up | Transcription factor HBI1 | HBI1 | brassinosteroid mediated signaling pathway |
| LOC103829630 | 1.37E-08 | Up | Cytochrome P450 734A1 | CYP734A1 | brassinosteroid metabolic process\|response to brassinosteroid |
| LOC103837116 | 0.01834 | Up | Probable membrane-associated kinase regulator 1 | MAKR1 | brassinosteroid mediated signaling pathway |
| LOC103837851 | 3.48E-93 | Up | Transcription factor HBI1 | HBI1 | brassinosteroid mediated signaling pathway |
| LOC103842602 | 2.19E-06 | Up | 3-epi-6-deoxocathasterone 23-monooxygenase | CYP90D1 | brassinosteroid biosynthetic process |
| LOC103843064 | 2.55E-70 | Up | AP2/ERF and B3 domain-containing transcription factor RAV1 | RAV1 | response to brassinosteroid |
| LOC103850156 | 0.007856 | Up | Transcription factor PRE1 | PRE1 | brassinosteroid mediated signaling pathway |
| LOC103850594 | 0.043448 | Up | Cytochrome P450 90A1 | CYP90A1 | brassinosteroid biosynthetic process |
| LOC103854158 | 0.049985 | Up | Probable indole-3-pyruvate monooxygenase YUCCA8 | YUC8 | brassinosteroid mediated signaling pathway |
| LOC103861022 | 6.26E-09 | Up | BES1/BZR1 homolog protein 3 | BEH3 | brassinosteroid mediated signaling pathway |
| LOC103862958 | 0.002011 | Up | Transcription factor PAR2 | PAR2 | brassinosteroid mediated signaling pathway |
| LOC103863753 | 2.06E-11 | Up | Probable serine/threonine-protein kinase PBL7 | PBL7 | brassinosteroid mediated signaling pathway |
| LOC103865793 | 2.91E-18 | Up | Protein RETICULATA-RELATED 5, chloroplastic | RER5 | response to brassinosteroid |
| LOC103870953 | 4.81E-30 | Up | Cytochrome P450 708A2 | CYP708A2 | brassinosteroid biosynthetic process |
| LOC103874079 | 1.46E-27 | Up | Transcription factor HBI1 | HBI1 | brassinosteroid mediated signaling pathway |
| LOC103874457 | 1.88E-10 | Up | Probable membrane-associated kinase regulator 1 | MAKR1 | brassinosteroid mediated signaling pathway |
| LOC103861043 | 3.63E-05 | Down | Abscisic acid 8'-hydroxylase 1 | CYP707A1 | brassinosteroid biosynthetic process |
| LOC103848793 | 0.004743 | Down | Probable serine/threonine-protein kinase At5g41260 | At5g41260 | brassinosteroid mediated signaling pathway |
| LOC103827806 | 0.026328 | Down | Abscisic acid 8'-hydroxylase 3 | CYP707A3 | brassinosteroid biosynthetic proces |
| LOC103851129 | 0.028929 | Down | Transcription factor MYB56 | MYB56 | brassinosteroid mediated signaling pathway |
