# Supplementary material for: Transcriptome analysis reveals a positive effect of brassinosteroids on the photosynthetic capacity of wucai under low temperature
Source: BMC Genomics. 2019 Nov 6;20:810. doi: 10.1186/s12864-019-6191-2 (PMC6836548; doi:10.1186/s12864-019-6191-2)
Supplement: Supplementary file 9 — Additional file 9: Table S5. Primers of verifying genes. [file 12864_2019_6191_MOESM9_ESM.docx]

| Table S5  Primers of verifying genes.   \| Gene name \| Primer name \| Primer sequence (5’->3’) \| \| --- \| --- \| --- \| \| *BnaActin* \| primer F \| TGGGTTTGCTGGTGACGAT \| \|  \| primer R \| TGCCTAGGACGACCAACAATACT \| \| LOC103861694 \| primer F \| GAGGTTTAGCTGGCGGATTG \| \|  \| primer R \| GAAGCGAATGTGACTCCAG \| \| LOC103831151 \| primer F \| GAGCTTCCCGACGAACAATC \| \|  \| primer R \| AGAACCACAAGCGAGAGAC \| \| LOC103828782 \| primer F \| CGCTCTCTCTTCCATCCTCC \| \|  \| primer R \| GCGCTTGGTCTTTTCGATCT \| \| LOC103829589 \| primer F \| CTTCTGCACGCCTCACTTG \| \|  \| primer R \| GGCAGTTCCGGTTCCTTTG \| \| LOC103832382 \| primer F \| AGATCAAGACCGACAAGCCT \| \|  \| primer R \| GTACACATCACCACTCGCT \| \| LOC103832805 \| primer F \| ACATTTGCTGCTCCTTTCACA \| \|  \| primer R \| AGGACCGAGTTTAGGCTTCT \| \| LOC103836470 \| primer F \| TCACTCCTACCGACAAGAAG \| \|  \| primer R \| TGCTTACCACCTTCATCTCCAT \| \| LOC103836610 \| primer F \| CTCTCCTTAACCGCCGCATC \| \|  \| primer R \| CTGTACTTTGCCACCCACGAG \| \| LOC103837695 \| primer F \| CACTGGCAAATTCTTAGTCCCT \| \|  \| primer R \| TGATCTCACCCACCGAAGC \| \| LOC103839762 \| primer F \| ACTCAGACCAAGCAAGAGACT \| \|  \| primer R \| GGTACGATGCTCTTAAACGGA \| \| LOC103843400 \| primer F \| TCGCTCACTTCCCAACTCC \| \|  \| primer R \| AAGTAATCTTCGGTCCTGC \| \| LOC103843499 \| primer F \| CGCCACTAGCAATCTCAATGTC \| \|  \| primer R \| AATGTTTGCTGTCGCCACTG \| \| LOC103844017 \| primer F \| CCGTCCAGATAGTGTGCAAAG \| \|  \| primer R \| CTGTATGGCGTGAAGTCTGTG \| \| LOC103844493 \| primer F \| CTCCTTATCCGCCGCATCGAC \| \|  \| primer R \| ACACTGCATTCAACGCCGAG \| \| LOC103855224 \| primer F \| CCGCATGATGTTCTTAGCTG \| \|  \| primer R \| CATTCAGTTCTTTGTTGGCTT \| \| LOC103855346 \| primer F \| TGACCAAGAGCAAACCCGAGA \| \|  \| primer R \| TTGACCATACCACACCCCTT \| \| LOC103871027 \| primer F \| CGTCGTCATAAGCCTCAGCAC \| \|  \| primer R \| ACTCCTTAGCACGATCATCTCC \| \| LOC103835217 \| primer F \| GCCTCAGTTCAATGGTCTTCG \| \|  \| primer R \| ATGGTGCTAGTCCGAATCTCC \| \| LOC103871224 \| primer F \| AACCCAAATCCACAAGAGCTG \| \|  \| primer R \| ATTTGCTCTGAAGTGGGTTGT \| \| LOC103872730 \| primer F \| TCTCCGATCTAACCAAACCGT \| \|  \| primer R \| CAGTCCCCTTAACCTCCATGT \| |
| --- | --- | --- | --- | --- | --- | --- | --- | --- | --- | --- | --- | --- | --- | --- | --- | --- | --- | --- | --- | --- | --- | --- | --- | --- | --- | --- | --- | --- | --- | --- | --- | --- | --- | --- | --- | --- | --- | --- | --- | --- | --- | --- | --- | --- | --- | --- | --- | --- | --- | --- | --- | --- | --- | --- | --- | --- | --- | --- | --- | --- | --- | --- | --- | --- | --- | --- | --- | --- | --- | --- | --- | --- | --- | --- | --- | --- | --- | --- | --- | --- | --- | --- | --- | --- | --- | --- | --- | --- | --- | --- | --- | --- | --- | --- | --- | --- | --- | --- | --- | --- | --- | --- | --- | --- | --- | --- | --- | --- | --- | --- | --- | --- | --- | --- | --- | --- | --- | --- | --- | --- | --- | --- | --- | --- | --- | --- | --- | --- | --- |
