# Supplementary material for: Transcriptome analysis reveals a positive effect of brassinosteroids on the photosynthetic capacity of wucai under low temperature
Source: BMC Genomics. 2019 Nov 6;20:810. doi: 10.1186/s12864-019-6191-2 (PMC6836548; doi:10.1186/s12864-019-6191-2)
Supplement: Supplementary file 10 — Additional file 10: Table S6. Primers of photosynthesis genes. [file 12864_2019_6191_MOESM10_ESM.docx]

Table S6

Primers of photosynthesis genes.

| Gene name | Primer name | Primer sequence (5’->3’) |
| --- | --- | --- |
| *BnaActin* | primer F | TGGGTTTGCTGGTGACGAT |
|  | primer R | TGCCTAGGACGACCAACAATACT |
| LOC103828782 | primer F | CGCTCTCTCTTCCATCCTCC |
|  | primer R | GCGCTTGGTCTTTTCGATCT |
| LOC103829589 | primer F | CTTCTGCACGCCTCACTTG |
|  | primer R | GGCAGTTCCGGTTCCTTTG |
| LOC103832382 | primer F | AGATCAAGACCGACAAGCCT |
|  | primer R | GTACACATCACCACTCGCT |
| LOC103832805 | primer F | ACATTTGCTGCTCCTTTCACA |
|  | primer R | AGGACCGAGTTTAGGCTTCT |
| LOC103836470 | primer F | TCACTCCTACCGACAAGAAG |
|  | primer R | TGCTTACCACCTTCATCTCCAT |
| LOC103837509 | primer F | TGCTGTATCTGCTTCCGCTA |
|  | primer R | ACAGTAAACGCTCTCGCAAA |
| LOC103837695 | primer F | CACTGGCAAATTCTTAGTCCCT |
|  | primer R | TGATCTCACCCACCGAAGC |
| LOC103839762 | primer F | ACTCAGACCAAGCAAGAGACT |
|  | primer R | GGTACGATGCTCTTAAACGGA |
| LOC103843400 | primer F | TCGCTCACTTCCCAACTCC |
|  | primer R | AAGTAATCTTCGGTCCTGC |
| LOC103843499 | primer F | CGCCACTAGCAATCTCAATGTC |
|  | primer R | AATGTTTGCTGTCGCCACTG |
| LOC103844017 | primer F | CCGTCCAGATAGTGTGCAAAG |
|  | primer R | CTGTATGGCGTGAAGTCTGTG |
| LOC103844493 | primer F | CTCCTTATCCGCCGCATCGAC |
|  | primer R | ACACTGCATTCAACGCCGAG |
| LOC103855224 | primer F | CCGCATGATGTTCTTAGCTG |
|  | primer R | CATTCAGTTCTTTGTTGGCTT |
| LOC103855346 | primer F | TGACCAAGAGCAAACCCGAGA |
|  | primer R | TTGACCATACCACACCCCTT |
| LOC103871027 | primer F | CGTCGTCATAAGCCTCAGCAC |
|  | primer R | ACTCCTTAGCACGATCATCTCC |
| LOC103833816 | primer F | ACGCTCAGATCGAAAAGACCA |
|  | primer R | CGCAGGTTTCTTCTCATCAC |
| LOC103835217 | primer F | GCCTCAGTTCAATGGTCTTCG |
|  | primer R | ATGGTGCTAGTCCGAATCTCC |
| LOC103871224 | primer F | AACCCAAATCCACAAGAGCTG |
|  | primer R | ATTTGCTCTGAAGTGGGTTGT |
| LOC103872730 | primer F | TCTCCGATCTAACCAAACCGT |
|  | primer R | CAGTCCCCTTAACCTCCATGT |
| LOC103873821 | primer F | TTCTCACCTCCAGCTACACAA |
|  | primer R | CCCATCAACATCATCACCACC |
