# Supplementary material for: Transcriptome analysis reveals a positive effect of brassinosteroids on the photosynthetic capacity of wucai under low temperature
Source: BMC Genomics. 2019 Nov 6;20:810. doi: 10.1186/s12864-019-6191-2 (PMC6836548; doi:10.1186/s12864-019-6191-2)
Supplement: Supplementary file 12 — Additional file 12: Table S8. Parameters derived from the OJIP transient for use in the current study. [file 12864_2019_6191_MOESM12_ESM.docx]

Table S8

Parameters derived from the OJIP transient for use in the current study.

| Parameters | Definition | |  |
| --- | --- | --- | --- |
| M_o_ | | The maximum rate of QA restored | |
| N | | The number of times QA restored from F_o_ to F_M_ | |
| V_j_ | | Relative variable fluorescence at the J-step (2 ms) | |
| F_v_/F_o_ | | The ratio of F_v_ to F_o_ (an expression of F_v_/F_M_) | |
| *ϕ*Do | | Quantum ratio for heat dissipation | |
| *ϕ*Eo | | Quantum yield for electron transport | |
| *ψ*_o_ | | The excitons occupied by the excitons captured in the reaction center to push electrons to other electron acceptors in the electron transport chain beyond QA to promote the ratio of QA reductive excitons | |
| RC/CS_o_ | | The number of active reaction centers per unit area | |
